# From local lesion to multisystem disease: integrated crosstalk among the gut microbiota, immune system, and host metabolism in endometriosis

**DOI:** 10.3389/fimmu.2026.1924352

**Published:** 2026-09-22

**Authors:** Yuewen Liao, Yuan Shi, Xiaoya Pang, Yuxin Yang, Xuanyi Chen, Zili Tang, Ping Xie, Xin Cao

**Affiliations:** 1 School of Acupuncture and Tuina, Chengdu University of Traditional Chinese Medicine, Chengdu, China; 2 The Affiliated Hospital of Chengdu University of Traditional Chinese Medicine, Chengdu University of Traditional Chinese Medicine, Chengdu, China; 3 Lanzhou Municipal Institute of Health Education, Lanzhou, China; 4 Key Laboratory of Acupuncture and Medicine Research (Nanjing University of Chinese Medicine), Nanjing University of Chinese Medicine, Nanjing, China; 5 Key Laboratory of Acupuncture for Senile Disease (Chengdu University of TCM), Ministry of Education, Chengdu University of Traditional Chinese Medicine, Chengdu, China

**Keywords:** endometriosis, gut microbiota dysbiosis, immunometabolism, oxidative stress, therapeutic strategies

## Abstract

Endometriosis (EMs) is a common estrogen-dependent inflammatory disease characterized by pelvic pain and infertility. Despite its substantial clinical burden, its etiology and pathogenesis remain incompletely resolved, and current management continues to be constrained by delayed diagnosis, limited therapeutic efficacy, and frequent disease recurrence. Growing evidence indicates that EMs is not merely a localized gynecological disorder but a disease state with multisystem pathophysiological features, a condition defined by crosstalk among the gut microbiota, the immune system, and host metabolism. This review synthesizes current evidence to establish a systems biology framework in which gut dysbiosis, immune dysfunction, and metabolic reprogramming are integrated as interconnected drivers of disease initiation, progression, and persistence. We first examine alterations in gut microbial composition and function, including estrogen metabolism dysregulation, expansion of opportunistic pathogens, and impaired intestinal barrier integrity, and discuss how these changes influence estrogen homeostasis, inflammatory signaling, and host-microbe interactions. We then explore how aberrations in innate and adaptive immunity promote immune evasion, chronic inflammation, angiogenesis, and ectopic lesion survival. Furthermore, we highlight the multifaceted remodeling of carbohydrate, lipid, and amino acid metabolism, and its link to cell proliferation and the immunosuppressive microenvironment. Particular emphasis is placed on the bidirectional interplay among microbial metabolites, immunometabolic signaling, oxidative stress, and tricarboxylic acid cycle (TCA) intermediates, all of which coalesce into a self-reinforcing pathological network. By integrating these domains, This review advances a unifying “microbiome-immune-metabolic” framework to elucidate the multisystem pathophysiological features of EMs, aiming to inspire the discovery of non-invasive biomarkers and the development of precision therapeutic strategies that target microbial, immune, and metabolic pathways.

## 1 Introduction

Endometriosis (EMs) is a chronic, estrogen-dependent inflammatory gynecological disorder characterized by the presence of endometrial-like tissue outside the uterine cavity (1, 2). The global burden of EMs is estimated to affect approximately 10% of female population of reproductive age, with an estimated 190–222 million individuals affected by EMs, which constitutes a major public health challenge (3, 4). Clinically, chronic pelvic pain and infertility are the hallmarks of EMs, which is frequently accompanied by fatigue, dyspareunia, gastrointestinal and urinary symptoms, and psychological distress (5, 6). EMs harms more than just fertility. Accumulating evidence indicates that EMs is associated with systemic health complications. It raises risks of adverse pregnancy events and long-term cardiovascular disease, further amplifying public health burden (7, 8). Though it is highly prevalent, patients frequently face diagnostic delays of 5 to 12 years, which accelerates disease progression (2, 9).

Initial investigations recognized EMs as the ectopic implantation of endometrium-like tissue. However, accumulating evidence demonstrates that EMs is characterized by intricate multisystem pathophysiological features encompassing signaling networks of the endocrine, immune, metabolic, and microbial systems (10, 11). Classical pathogenetic theories of retrograde menstruation, coelomic metaplasia, and embryonic Müllerian duct remnants failed to explain why only a subset of women develop persistent, progressive lesions. This limitation has redirected research priorities toward host susceptibility and widespread systemic biological perturbations (10, 12).

Recent studies showed that gut microbiota dysbiosis, particularly alterations within the estrobolome, is associated with changes in estrogen bioavailability and inflammatory tone, thereby shaping immune cell activation and metabolic reprogramming in EMs (13, 14). Additionally, deep immunophenotyping studies and genetic causal analyses have identified systemic immune dysregulation, encompassing mononuclear phagocytes, natural killer (NK) cells, and diverse lymphocyte subsets, as an important feature associated with disease susceptibility and progression (15, 16). Advances in high-throughput sequencing and microbiome research further suggest that gut microbiota alterations may be associated with endotoxemia, alter estrogen recirculation, and activate immune system in EMs initiation and progression (13, 17). These findings further corroborate that EMs manifests multisystem pathophysiological features. Gut microbiota dysfunction can compromise immune function, induce aberrant metabolic reprogramming, and disrupt host-microbiota crosstalk, thereby driving persistent chronic inflammation and the maintenance of ectopic lesions (18, 19). Accordingly, an integrative systems biology framework is critical for exploring the interactive relationships among these biological processes during EMs lesion development and maintenance.

While accumulating evidence has independently associated gut microbiota dysbiosis, immune dysregulation, and metabolic reprogramming with the pathophysiology of EMs, these biological domains have largely been investigated in isolation. Consequently, how alterations in the gut microbiota, immune responses, and metabolic programming interact across multiple biological levels remains incompletely understood (20–22). To address this gap, this review adopts a systems biology perspective to integrate current evidence on microbiota, immune and metabolic interactions in EMs. Rather than proposing a definitive causal sequence, we summarize the multidirectional crosstalk among these interconnected processes and discuss how they may contribute to lesion establishment, disease progression, and therapeutic heterogeneity. This review innovatively integrates the triple interactive network of gut microbiota, immunity, and metabolism as a framework for understanding chronic inflammation and lesion persistence in EMs, thereby informing future research directions and therapeutic innovation (13, 23).

This narrative review is based on targeted literature searches performed in the PubMed and Web of Science databases. Searches covered publications from database inception through July 2026. The search was restricted to articles published in English. Search terms were combined using the Boolean operators AND OR according to the main themes of the review. The primary search terms included: “endometriosis”, “gut microbiota”, “dysbiosis”, “intestinal barrier”, “immune dysregulation”, “oxidative stress”, “tricarboxylic acid cycle”, “estrobolome”, “natural killer cell”, “macrophage polarization”, “T helper 17 cell”, “regulatory T cell”, “immune checkpoint”, “metabolic reprogramming”, “glycolysis”, “lipid metabolism”, “sphingolipid”, “cholesterol metabolism”, “carnitine”, “amino acid metabolism”, “tryptophan”, “glutamine”, “reactive oxygen species”, “Nrf2”, “succinate”, “short-chain fatty acids”, “indole metabolites”, “biomarkers”, “metabolomics”, “microbiome”, “dietary intervention”, “Mediterranean diet”, “ketogenic diet”, “low FODMAP diet”, “probiotics”, “fecal microbiota transplantation”, “immunotherapy”, “hormonal therapy”, and “metabolic therapy”. We prioritized peer-reviewed human studies, clinical trials, systematic reviews, meta-analyses, and key mechanistic investigations directly relevant to the “gut microbiota–immunity–metabolism” axis in EMs. Given the limited direct causal evidence from human cohorts and substantial heterogeneity across published findings, animal models, *in vitro* cell-based experiments, and nonhuman primate studies were also included when they provided critical mechanistic insights into pathogenesis or therapeutic mechanisms unavailable from human investigations. Literature screening was performed based on relevance to the core mechanistic, diagnostic, and therapeutic themes of this narrative review, rather than predefined systematic-review eligibility criteria. Where conflicting results were reported across studies, we appraised study quality, sample size, cohort characteristics, and methodological consistency, and interpretations prioritized large well-characterized cohorts, independently replicated studies, and meta-analyses.

## 2 Pathophysiology of endometriosis: a multisystem pathophysiological perspective

### 2.1 Traditional local pathogenesis theories of endometriosis

Sampson’s theory of retrograde menstruation remains the cornerstone of EMs etiology, proposing that viable endometrial fragments reflux through the fallopian tubes and implant ectopically (24, 25). This classic mechanical implantation theory has long served as the core local pathogenetic mechanism to explain the occurrence of pelvic endometriotic lesions. However, retrograde menstruation is a common physiological phenomenon that occurs in most menstruating women, which means this single local mechanical mechanism cannot fully explain the selective onset of EMs in specific populations, nor can it account for the formation of extrapelvic lesions (26, 27). The limitations of the traditional local theory indicate that additional regulatory factors beyond mechanical implantation may be involved in the progression of EMs, supporting investigation of multisystem pathophysiological factors.

### 2.2 Beyond the local lesion paradigm

The discovery of endometriotic lesions in distant organs fundamentally challenges the traditional purely mechanical local pathogenesis model, confirming the existence of systemic permissive factors that dominate disease occurrence and development (24, 26). Multiple lines of emerging evidence support the multisystem pathological characteristics of EMs. Mechanistically, gut microbiota dysbiosis modulates the pelvic microenvironment through microbial-derived components and metabolites, including lipopolysaccharide (LPS), short-chain fatty acids (SCFAs), and indole derivatives. Upon entering the systemic circulation, these bioactive mediators regulate immune and metabolic homeostasis (28–31). Furthermore, alterations in microbial communities reported in refluxed menstrual effluent have been proposed to interact with immune and metabolic processes, providing a potential link between retrograde menstruation and chronic immune-metabolic dysregulation; however, this relationship remains to be established in humans (25, 32). Importantly, most microbial signatures detected in human menstrual blood and peritoneal fluid primarily reflect resident genital tract microbiota, rather than viable gut-derived bacteria (32, 33). Collectively, gut microbiota exert their pathogenic roles in EMs as a systemic immunometabolic regulator via microbial metabolites and bioactive products, instead of relying on the direct translocation of viable gut bacteria into the pelvic cavity. Etiologically, EMs susceptibility arises from the interplay of multiple systemic determinants, including genetic, environmental, hormonal, and developmental factors (34, 35). For example, susceptibility loci such as *Interleukin 1 alpha* (*IL1A*) and *Growth regulating estrogen receptor Binding 1* (*GREB1*) regulate inflammatory and immune pathways that influence ectopic endometrial implantation and lesion progression (32, 36, 37). Environmentally, endocrine-disrupting chemicals such as bisphenol A and phthalates disrupt hormonal balance and are associated with an increased risk of EMs (38, 39). Further studies indicate that iron and environmental metal accumulation within human ovarian endometriotic lesions may constitute an independent pathogenic mechanism of EMs (40). Hormonal dysregulation represented by hyperestrogenism and progesterone resistance is associated with lesion proliferation and persistent progression and may contribute to disease development (36, 41). In addition, early-life developmental exposures such as low birth weight and *in-utero* diethylstilbestrol (DES) exposure have been associated with long-term susceptibility to EMs (42, 43). These multifactorial systemic disturbances break through the limitations of local lesion theory and reshape the systemic pathogenic cognition of EMs.

### 2.3 Perturbed gut microbiota, immune, and metabolic homeostasis: a multisystem pathophysiological framework

From a systems-biology perspective, EMs may be conceptualized as a disease exhibiting multisystem pathophysiological features in which aberrant ectopic tissue repair is sustained by coordinated interactions among the gut microbiota, the immune system, and host metabolism. Rather than representing independent pathological processes, these systems shape the permissive microenvironment required for lesion establishment, progression, and persistence (44). The initial pathological event is thought to arise following retrograde menstruation, when physiological wound-healing programs are inappropriately activated at extrauterine sites. Instead of resolving tissue injury, these repair responses enable ectopic endometrial cells to acquire enhanced adhesive, migratory, and invasive capacities, thereby promoting lesion establishment rather than passive implantation (24, 38, 45). Hyperresponsive endometrial stromal cells compromise tissue integrity, whereas sustained *Vascular endothelial growth factor* (*VEGF*)-driven angiogenesis provides the vascular support associated with lesion growth, survival, and deep infiltration (46, 47). This abnormal repair process is continuously amplified and maintained by interactions among the three regulatory systems. Gut dysbiosis contributes to intestinal barrier disruption and initiates systemic inflammatory responses, which subsequently promote immune dysregulation and persistent cytokine production. In parallel, oxidative stress, redox imbalance, and epigenetic alterations are associated with metabolic reprogramming, thereby reinforcing immune dysfunction and establishing a pro-fibrotic, pro-angiogenic microenvironment that favors lesion progression (41–43).

## 3 Gut microbiota dysbiosis: a multisystem contributor to endometriosis

### 3.1 Mechanisms and driving factors of endometriosis

The gut-immunity-metabolism axis constitutes the overarching theoretical framework of this review, which posits that gut microbiota systematically regulates immune differentiation, inflammatory signaling, and whole-body metabolic homeostasis. In EMs, the gut–uterus axis provides a disease-specific mechanistic framework for understanding how gut-derived signals modulate pelvic microenvironmental homeostasis. This axis functions through two independent pathogenic pathways. Pathway 1 (metabolite-mediated pathway): Gut microbiota-derived metabolites gain access to the systemic circulation and disseminate to multiple target organs, including the reproductive tract, adipose tissue, and central nervous system, thereby executing systemic immune and metabolic regulation. Pathway 2 (translocation-mediated pathway): Intestinal barrier dysfunction allows microbial products and bacterial components to enter systemic circulation, exerting pathological impacts on the pelvic and peritoneal cavities. Additionally, mesenteric lymphatic drainage serves as a direct local route for gut-derived mediators to affect peritoneal tissues.

The gut microbiome acts as a central component of the proposed axis. The oral microbiome serves as an upstream source of microbial translocation and a promising non-invasive diagnostic biomarker, yet it is not a central pathogenic driver. By contrast, the reproductive tract microbiome functions as a local pathological effector and a site-specific microbial biomarker for EMs.

### 3.2 Characteristic microbial signatures of endometriosis dysbiosis

Accumulating evidence suggests that gut microbial composition is altered in EMs; however, the reported microbial profiles remain considerably heterogeneous across studies (48). This variability likely reflects differences in sampling site (stool versus mucosal microbiota), disease subtype (peritoneal, ovarian, or deep infiltrating EMs), menstrual cycle phase, antibiotic exposure, dietary habits, sequencing platforms, and control population selection. Consequently, despite increasing evidence for gut microbial dysbiosis, no universal EMs-associated microbial signature has yet been established. Against this heterogeneous background, alterations in microbial diversity represent one of the most frequently investigated features. Several clinical studies have reported reduced α-diversity in women with EMs, suggesting diminished microbial richness and evenness that may reflect a less resilient intestinal ecosystem with impaired immune homeostasis (23, 49). Likely, changes in β-diversity have been observed in multiple cohorts, indicating disease-associated shifts in overall community composition rather than random microbial variation (50). Nevertheless, these diversity patterns are not consistently reproduced across all studies and, at present, should be regarded as indicators of microbial instability rather than sufficiently robust biomarkers for clinical diagnosis or a definitive microbial signature of EMs. Taxonomic alterations have also been widely reported, although their interpretation requires caution (51). While several studies describe changes in the relative abundance of bacterial groups such as Lactobacillus, Proteobacteria, and members of Firmicutes, these findings vary substantially between cohorts (48). Moreover, describing entire bacterial phyla as uniformly “pro-inflammatory” is overly simplistic because these taxonomic groups encompass metabolically and immunologically diverse species with distinct functional properties. Current sequencing studies also often lack sufficient species- or strain-level resolution to determine which microorganisms are functionally relevant to disease pathogenesis. Therefore, taxonomic shifts should be interpreted as reflecting alterations in microbial community structure rather than direct evidence that specific bacterial taxa universally drive EMs. Murine and nonhuman primate studies have demonstrated that experimentally induced EMs can alter gut microbial composition and that microbiota manipulation may influence lesion progression, supporting biological crosstalk between gut microbial ecology and disease development (50, 52, 53).

### 3.3 Pathogenic microbial expansion and phenotypic alteration in endometriosis

Recent studies have highlighted the pathogenic overgrowth of Gram-negative bacteria such as *Escherichia coli* (*E. coli*) and *Shigella* in EMs cohorts, suggesting a direct link between these microbes and disease progression. This dysbiosis is associated with a chronic inflammatory state, contributing to the pathophysiology of EMs. Specifically, *E. coli* has been found to be significantly more abundant in women with EMs compared to controls, supporting its role in disease development (54). The presence of *Shigella* has similarly been implicated, suggesting that these bacteria may act as key players in the immune activation and inflammatory responses seen in EMs (55). Studies have shown that such bacterial overgrowth can alter the gut microbiome’s composition, exacerbating local and systemic inflammation (48). Moreover, *E. coli* and *Shigella* can influence estrogen metabolism and immune cell function, which may further promote the persistence and growth of endometriotic lesions (17, 56). Khan and colleagues demonstrated that Gram-negative bacteria, predominantly *E. coli*, ascend from the vagina and contaminate menstrual blood, acting as a sustained pelvic reservoir of LPS (57). Patients with EMs exhibit significantly higher *E. coli* colony counts and a four- to six-fold increase in menstrual LPS levels compared with healthy controls (58). Mechanistically, LPS binds to and activates Toll-like receptor 4 (TLR4) on pelvic macrophages, triggering the downstream *nuclear factor kappa-light-chain-enhancer of activated B cells* (*NF-κB*) signaling pathway. This process robustly induces the secretion of pro-inflammatory cytokines, including IL-6, TNF-α, and IL-1β, which initiates pelvic inflammatory responses and promotes endometrial cell proliferation (55). The persistent LPS-TLR4 signaling cascade further forms a self-amplifying positive feedback loop that facilitates the progressive development of ectopic lesions (59). These findings underscore the importance of Gram-negative bacterial abundance in shaping the microbial landscape in EMs, potentially offering new therapeutic avenues aimed at restoring microbiome balance to mitigate disease symptoms (60, 61).

### 3.4 The estrobolome dysregulation in endometriosis

Beyond compositional shifts, gut microbiota dysbiosis in EMs has been increasingly conceptualized through the lens of the estrobolome, defined as the aggregate of enteric bacterial genes involved in estrogen metabolism and deconjugation. The best-characterized mechanism by which gut microbes govern systemic estrogen bioavailability relies on β-glucuronidase. This intestinal enzyme deconjugates estrogen-glucuronide complexes, liberating biologically active estrogens for intestinal reabsorption and further regulating enterohepatic estrogen circulation and overall systemic estrogen homeostasis. However, emerging evidence has validated that microbial steroid sulfatase (STS) may also contribute to estrogen metabolism (62). Estrone sulfate (E1S), the most abundant circulating estrogen in humans, can be rapidly converted into biologically active estrogen via STS-mediated catalysis. Since most estrogens secreted into bile exist in sulfated forms, gut microbial STS is functionally more crucial than β-glucuronidase in modulating estrogen recycling. Moreover, other microbial enzymes such as β-glucosidase are speculated to participate in this metabolic process, yet their exact functional roles in human estrogen metabolism remain poorly elucidated (63). Collectively, the estrobolome should be redefined as a functionally heterogeneous and interactive microbial network, rather than a single-enzyme regulatory system. Currently, the Human Microbiome Project’s gastrointestinal dataset identifies 279 unique gut microbial β-glucuronidase proteins, classified into six structural categories (64). Dysregulation of this estrobolome has been implicated in estrogen-dependent disorders, including EMs, where persistent hyperestrogenism fuels lesion survival and inflammatory signaling (55). Recent case-control analyses demonstrate that women with EMs exhibit elevated levels of circulating and fecal estrogen metabolites despite minimal differences in overall microbial diversity, underscoring a functional rather than purely taxonomic alteration of the gut microbiome (65). Parallel evidence from endometrial dysbiosis studies suggests that overrepresentation of estrogen-metabolizing bacteria enhances local and systemic inflammatory milieus while suppressing anti-inflammatory immune cell populations (66). Notably, most current human microbiome studies are observational and vulnerable to the following confounding factors: age, body mass index (BMI), menstrual cycle phase, use of hormonal contraception, gonadotropin-releasing hormone (GnRH) therapy, antibiotic use, use of non-steroidal anti-inflammatory drugs (NSAIDs), diet, constipation or irritable bowel syndrome (IBS), geographic region, participant ancestry, disease stage, lesion subtype, prior surgical history, infertility treatment, and sample collection or storage procedures (67). Although animal experiments and cellular investigations have yielded preliminary mechanistic evidence, reported gut microbiota-EMs associations to date remain largely correlative. Observed microbial alterations cannot yet be directly attributed to the disease itself, and high-quality causal evidence remains to be established (68, 69).

### 3.5 Intestinal barrier dysfunction: the “leaky gut” hypothesis and systemic inflammatory initiation in endometriosis

An emerging mechanistic axis linking gut microbiota dysbiosis to EMs centers on increased intestinal permeability and the consequent translocation of microbial antigens into the systemic circulation. Multiple studies report that dysbiosis-associated disruption of epithelial tight junctions facilitates the leakage of bacterial components, notably LPS derived from Gram-negative bacteria, into portal and systemic blood flow, thereby amplifying low-grade chronic inflammation (23, 49). Elevated circulating endotoxins can activate innate immune pathways, including TLR4 signaling, promoting proinflammatory cytokine release such as TNF-α and IL-6, which are consistently increased in EMs patients (19, 70). Experimental and clinical data further suggest that compromised gut barrier integrity impairs immune surveillance and favors ectopic endometrial cell survival, adhesion, and angiogenesis within the peritoneal cavity (19, 71). Notably, dysregulated microbial metabolites, including reduced SCFAs, may exacerbate epithelial dysfunction by weakening mucosal energy supply and anti-inflammatory signaling (71). In parallel, altered gut-immune crosstalk has been linked to neuroimmune modulation and pain sensitization, suggesting that intestinal permeability may represent a potential contributor to systemic manifestations rather than a purely localized gastrointestinal phenomenon (70). Collectively, these findings support a systems-biology framework in which gut barrier breakdown serves as a critical conduit connecting microbial dysbiosis to immune activation, metabolic disturbance, and the persistent inflammatory milieu characteristic of EMs. Whether this relationship is causal in humans remains to be established. Key features of gut microbiota dysbiosis and their systemic implications in EMs are summarized in **Table 1**.

**Table 1:** Key features of gut microbiota dysbiosis and their systemic implications in endometriosis.

Dysbiosis feature	Key microbial taxa/function	Immediate biological effect	Systemic immunometabolic consequence	Relevance to endometriosis	Evidence Level	Ref
Reduced a-diversity	Depletion of Lactobacillus spp.	Diminished microbial ecosystem resilience	Impaired immune tolerance and heightened systemic inflammation	Promotes chronic inflammation and lesion persistence	3b (multiple case-control studies, inconsistent results; confounders: cycle phase, diet, antibiotics, platform)	(48)
Altered β-diversity	Enrichment of Proteobacteria	Disease-associated restructuring of the microbial community	Sustained activation of innate immunity	Contributes to systemic inflammatory priming	3b (multiple case-control studies, relatively consistent; confounders: subtype, sample site, medications)	(51)
Pathogenic overgrowth	Escherichia coli,Shigella	Increased LPS production	TLR4-mediated release of pro-inflammatory cytokines (TNF-α, IL-6)	Promotes angiogenesis and survival of ectopic lesions	4 (human case-control + animal models; enrichment observed but causality unconfirmed; confounders: contamination, infections, antibiotics)	(54)
Estrobolome dysregulation	β-glucuronidase- producing bacteria	Enhanced deconjugation and reabsorption of estrogens	Systemic estrogen excess	Fuels estrogen-dependent lesion growth	4 (human observational; β-GLC mechanism well-established in microbiome field, but EMs-specific evidence limited; confounders: diet, antibiotics, hormones)	(65)
Increased intestinal permeability	Disruption of tight junctions	Translocation of microbial products	Endotoxemia and impaired immune surveillance	Facilitates lesion adhesion and immune evasion	5 (predominantly animal models; indirect human evidence; direct measurements scarce; confounders: IBS, diet, NSAIDs)	(70)
Reduced SCFAs production	Depletion of butyrate-producing bacteria	Impaired epithelial energy supply and barrier maintenance	Worsened barrier dysfunction and sustained local inflammation	Contributes to chronic pain and inflammatory milieu	5 (strong in animal models; weak in human cohorts; confounders: fiber intake, transit time, microbiota composition)	(71)

Evidence levels were classified according to clinical trial and translational research grading standards. Level 3b: Individual high-quality case-control studies. Level 4: Case series, case-control, or cohort studies with high risk of bias. Level 5 (Lowest): Expert opinion, bench research, or mechanistic.

## 4 Immunoregulatory dysfunction in endometriosis: potential mediators of multisystem pathophysiological features

Under physiological conditions, retrograde menstrual debris entering the peritoneal cavity is rapidly cleared by resident local immune cells. In human studies, patients with EMs have been reported to exhibit functional alterations across both innate and adaptive immune compartments. These alterations may weaken immune surveillance and may be associated with the survival, implantation, and persistence of ectopic endometrial cells (28).

### 4.1 Innate immune system dysfunction in endometriosis

#### 4.1.1 Natural killer cell dysfunction in endometriosis

NK cells are a critical component of the innate immune system, eliminating ectopic endometrial cells through cytotoxic activity and thereby preventing their ectopic implantation (29). In patients with EMs, peripheral NK (pNK) cells exhibit significantly increased expression of inhibitory killer immunoglobulin-like receptors (KIRs), particularly KIR2DL1, which contains immunoreceptor tyrosine-based inhibitory motifs (ITIMs) (72, 73). A hyperestrogenic microenvironment serves as a core pathogenic driver of natural killer (NK) cell dysfunction in EMs. Clinical studies have confirmed that patients with stage III/IV advanced EMs present markedly increased plasma estradiol (E2) levels, which are strongly negatively correlated with reduced NK cell cytotoxic activity (74). Excessive estrogen exposure also interferes with the normal differentiation and functional maturation of NK cells (75). Consistent with these findings, pharmacological estrogen suppression via GnRH analogues effectively restores the quantity and bioactivity of NK cells, verifying the direct inhibitory impacts of estrogen on NK cell function (76). In contrast to the immunosuppressive effects of estrogen, progesterone exerts protective regulatory properties against immune dysfunction. A recent clinical study demonstrated that the progestin agent dienogest significantly enhances NK cell immune activity (77). Progesterone receptors (PR) are abundantly expressed on both peripheral circulating and endometrial-resident NK cells, indicating that progesterone modulates NK cell function through a direct receptor-binding mechanism (76). In the ovariectomy-plus-endometriosis (OVX+END) mouse model, in which mice are ovariectomized and then surgically implanted with endometrial tissue to study endometriosis under controlled hormone conditions, gut dysbiosis-associated metabolic alterations, including elevated tricarboxylic acid (TCA) metabolites and reduced butyrate, have been implicated in NK cell dysfunction, potentially affecting cytotoxic activity and receptor profiles (36). Overall, these findings indicate that gut dysbiosis and NK cell alterations coexist and are functionally linked in EMs. Moreover, microbial metabolite changes, together with estrogen metabolome disturbances, may represent key upstream factors contributing to NK cell dysfunction.

#### 4.1.2 Transcriptional and functional states of macrophages in endometriosis

Macrophages exhibit profound functional heterogeneity and serve as central regulatory cells within the peritoneal microenvironment of EMs. Although the canonical M1/M2 paradigm provides a fundamental conceptual framework for interpreting macrophage activation, it fails to fully recapitulate the intricate phenotypic diversity of macrophages in human pathological conditions. Accumulating evidence demonstrates that macrophage phenotypes exist along a dynamic continuum, collectively modulated by multiple intrinsic and extrinsic factors, including cellular origin, tissue microenvironment, inflammatory and hormonal signaling, hypoxia, extracellular matrix composition, and metabolic cues. Consequently, macrophages residing in endometriotic lesions do not conform to discrete M1 or M2 subsets; instead, they display diverse transcription profiles and functional phenotypes specifically shaped by the local lesional microenvironment (78).

Cellular ontogeny is a critical determinant of macrophage functional behaviors in EMs. Peritoneal macrophages consist of two major populations: embryonic-derived tissue-resident macrophages and monocyte-derived macrophages recruited during postnatal inflammatory activation. Large peritoneal macrophages (LpMs) represent the dominant resident macrophage subset in the peritoneal cavity, primarily established during embryonic development and partially replenished by circulating monocytes in adulthood. In the context of EMs, lesion-associated macrophages originate from multiple sources, including eutopic endometrial macrophages, resident peritoneal LpMs, and circulating monocytes recruited to ectopic microenvironments (79). Functional validation via ontogenetic intervention further confirms the decisive role of cellular origin: replacement of embryonic-origin LpMs with monocyte-derived LpMs significantly attenuates ectopic lesion formation, indicating that macrophage ontogeny modulates host susceptibility to EMs pathogenesis (79).

Single-cell transcriptomic studies have further validated that EMs-associated macrophages comprise multiple functionally distinct subpopulations, rather than following a simplified M1/M2 binary classification. In preclinical murine models, Henlon and colleagues identified two core lesion-resident macrophage subsets. Tumor-associated macrophage-like cells facilitate the transcription of *COL1A1* and *TGFB1* in endometrial stromal cells and promote endothelial angiogenic activation; by contrast, scar-associated macrophages exhibit pro-fibrotic and matrix-remodeling phenotypes (80). Conversely, a pro-resolving LpM subset characterized by lipid remodeling and enhanced cholesterol efflux exerts protective effects. Pharmacological activation of this lipid metabolic pathway via ApoE mimetics reduces lesion volume and alleviates local fibrosis, confirming that specific macrophage subpopulations mediate protective rather than pathogenic effects in EMs.

Human single-cell sequencing data further uncover prominent macrophage heterogeneity in ovarian endometriotic lesions. Tan et al. categorized ovarian lesion macrophages into five distinct subsets: MΦ1-LYVE1, MΦ2-peritoneal, MΦ3-APOE, MΦ4-infiltrated, and MΦ5-activated (80). Perivascular MΦ1-LYVE1 macrophages, a tissue-resident subset characterized by LYVE1 expression and homeostatic functions, dominate ovarian endometriotic lesions, while MΦ4-infiltrated macrophages are rarely detected in ovarian endometriomas. This distinct distribution pattern suggests that the local microenvironment differentially regulates the recruitment, retention, and activation of monocyte-derived macrophages. Additionally, multiple single-cell analyses have identified unique macrophage populations, including C1q-expressing macrophages, hyperactivated macrophages, NRN1-positive macrophages, and myofibroblast-like macrophages, further illustrating the robust phenotypic plasticity of macrophages in EMs (78).

Spatial localization constitutes an additional layer of macrophage functional heterogeneity in endometriotic lesions. Burns et al. demonstrated that epithelial-macrophage signaling activity within ectopic lesions is 3.7-fold higher than that in paired eutopic endometrial tissues from the same patients (81). Superficial lesional epithelial cells modulate local inflammatory signaling and drive tissue repair-related functional programming in macrophages, while complement component 3 (C3) acts as a key mediator of immune crosstalk within endometriotic microenvironments (81). Spatial transcriptomic profiling also confirms the colocalization of M2-like resident macrophages and fibrotic regions in ectopic lesions. These findings indicate that macrophage functionality cannot be interpreted independently of anatomical location and adjacent cellular components. Instead, macrophage phenotypic states are dynamically and persistently shaped by reciprocal interactions with epithelial cells, stromal cells, endothelial cells, extracellular matrix, and other immune populations within lesions.

Macrophage functional phenotypes are tightly coupled to cellular metabolic reprogramming. Metabolic remodeling is not merely a downstream consequence of macrophage activation but an upstream driver that actively determines macrophage polarization and effector functions. Alterations in glycolysis, lactate metabolism, lipid turnover, cholesterol efflux, mitochondrial respiration, amino acid metabolism, iron metabolism, and hypoxia-responsive signaling collectively regulate macrophage-mediated inflammation, tissue remodeling, angiogenesis, and fibrogenesis (80, 82, 83). Conversely, inflammatory cytokines, hypoxic stress, extracellular metabolites, and stromal cell-derived signals reshape macrophage metabolic profiles, forming a bidirectional regulatory loop between local metabolic microenvironments and immune function (84, 85).

Hormonal immune modulation serves as the core endocrine basis for persistent inflammation and immune tolerance in endometriotic microenvironments. Sex steroid hormones are master regulators of macrophage phenotypic and functional remodeling. Estrogen modulates macrophage recruitment, polarization, and effector function through multiple signaling cascades. Mechanistically, estrogen activates *NF-κB* via estrogen receptor beta (ERβ), upregulates stromal CCL2 expression, and enhances macrophage recruitment to ectopic lesions (86). 17β-estradiol (E2) further promotes M2 macrophage polarization by activating STAT3 and P38 MAPK signaling pathways (87). Functionally, estrogen stimulates macrophage-derived HGF secretion to facilitate lesion growth and modulates macrophage immune responses via G protein-coupled estrogen receptor (GPER) upregulation (88, 89). In contrast, progesterone exerts protective, anti-inflammatory effects. Peritoneal macrophages constitutively express PR, and progesterone suppresses endometrial MIF secretion, thereby markedly reducing macrophage infiltration in murine EMs models (90). However, pervasive progesterone resistance in EMs patients impairs the physiological protective regulatory effects of progesterone on macrophages (91).

#### 4.1.3 Dendritic cell maturation defects in endometriosis

Dendritic cells (DCs) bridge innate and adaptive immune signaling cascades, yet their maturation program is severely disrupted in EMs. This maturation block abrogates their ability to initiate antigen-specific adaptive immune responses targeting ectopic endometrial cells (92). Impaired DC antigen presentation weakens peritoneal clearance of retrograde endometrial debris during menstruation, creating permissive conditions for ectopic lesion establishment (93).

#### 4.1.4 Myeloid-derived suppressor cell expansion in endometriosis

Myeloid-derived suppressor cell (MDSC) expands robustly within lactate-enriched peritoneal microenvironments, potently suppressing T-cell effector responses. MDSC accumulation generates a niche characterized by chronic inflammation, enhanced angiogenesis, and progressive tissue fibrosis, all of which support lesion survival and expansion (22). Coordinated dysfunction across all these innate immune cell subsets collectively shapes a microenvironment biased toward sustained inflammation and tissue remodeling rather than targeted immune clearance, laying a foundational immunological framework for early ectopic lesion seeding and survival.

#### 4.1.5 Mast cell, neuroimmune interactions in pain sensitization

Elevated peritoneal neutrophil counts and mast cell-mediated neuroinflammation amplify local tissue injury and peripheral pain sensitization (94). C3 serves as a critical pathogenic mediator in EMs. In response to persistent pro-inflammatory stimulation within the pelvic microenvironment, C3 robustly induces mast cell activation, augments local inflammatory cascades, and ultimately propels endometriotic disease progression (95). In human studies, mast cell numbers are significantly elevated in ectopic lesions, particularly in close proximity to nerve fibers (96, 97). Mast cells are key mediators of neuroinflammation and pain sensitization in EMs through the release of histamine, nerve growth factor (NGF), tryptase, and substance P (96). NGF promotes the sprouting and survival of sensory nerve endings within ectopic lesions, leading to hyperinnervation and heightened pain sensitivity. Histamine and tryptase directly sensitize peripheral nociceptors, lowering the threshold for pain transmission.

### 4.2 Adaptive immune system dysregulation in endometriosis

#### 4.2.1 T helper 17/regulatory T cell imbalance as the core mechanism of immune dysregulation in endometriosis

T lymphocytes serve as central mediators of adaptive immunity and are categorized into CD4^+^ and CD8^+^ subsets. CD4^+^ T cells are further subdivided into four functionally distinct populations: T helper 1 (Th1), T helper 2 (Th2), T helper 17 (Th17), and regulatory T cells (Tregs). Th1 and Th17 cells exert pro-inflammatory effector functions, whereas Th2 cells and Tregs mediate anti-inflammatory signaling.

Under physiological conditions, the Th1/Th2 balance is driven by pro-inflammatory signals (IL-12/TNF-α) to promote Th1 differentiation and maintain cellular immunity. In EMs, however, despite elevated Th1 cells and IL-12, Th2-type cytokines are also significantly upregulated, resulting in a state where Th1 and Th2 coexist, though with a shift toward Th2 predominance (98, 99). While pro-inflammatory responses persist throughout the disease course, anti-inflammatory factors gradually gain dominance with disease chronicity (100).

Simultaneously, aberrantly activated Th17 cells are correlated with amplified inflammatory signaling within the peritoneal niche. IL-17, the primary effector cytokine secreted by Th17 cells, is elevated in both plasma and peritoneal fluid of EMs patients. Human tissue analyses and *in vitro* studies suggest that IL-17 may contribute to disease progression by stimulating angiogenesis and inducing secondary pro-inflammatory cytokine release (101, 102). A unique IL-10-expressing Th17 subpopulation has also been detected within ectopic lesions; its abundance increases with disease stage following stimulation by IL-27 secreted by macrophages and endometrial stromal cells, further amplifying local inflammatory cascades (103).

Tregs dysfunction is associated with the characteristic immunosuppressive microenvironment of EMs. Clinical patient cohorts and baboon preclinical models demonstrate expanded FOXP3^+^ Tregs populations within ectopic lesions, while peripheral blood and peritoneal fluid exhibit reduced Tregs frequencies. In contrast, forkhead box protein P3 (FOXP3) expression is elevated in eutopic endometrium during the follicular phase of the menstrual cycle (104, 105). A case-control study of 55 EMs patients demonstrated that *FOXP3* expression was significantly higher in deep rectosigmoid endometriotic lesions than in eutopic endometrium, and that *FOXP3* expression in the eutopic endometrium of these patients was associated with chronic pelvic pain (106). These observations indicate that Tregs dysfunction may modulate both systemic and local inflammatory signaling and may be implicated in the pathophysiology of EMs.

Disrupted Th17/Treg homeostasis has been correlated with EMs onset and progression. In human cohort studies, untreated patients with EMs exhibit significantly reduced peripheral Tregs counts and expanded Th17-cell populations, resulting in a sharp decline in the Treg/Th17 ratio, which reflects a persistent systemic inflammatory state (107). This suppressed Treg/Th17 ratio has been independently validated in non-human primate disease models (104). Meanwhile, evidence indicates that gut dysbiosis leads to reduced SCFAs levels, thereby attenuating Tregs induction and Th17 suppression, thus exacerbating the Th17/Treg imbalance and promoting the survival, invasion, and lesion formation of ectopic endometrial tissue (108).

#### 4.2.2 CD8^+^ cytotoxic T cell exhaustion and immune checkpoints in endometriosis

CD8^+^ cytotoxic T cell (CD8^+^ T cell) lymphocytes are essential for immune surveillance against ectopic endometrial cells. In healthy endometrium, CD8^+^ T cells can suppress ectopic cell survival through cytotoxic activity (109). In EMs patients, however, CD8^+^ T cells exhibit a dysfunctional state characterized by exhaustion and impaired effector function. In human studies, CD8^+^ T cells in the peritoneal fluid and peripheral blood of EMs patients display upregulated expression of inhibitory immune checkpoint molecules, including programmed cell death protein 1 (PD-1), Cytotoxic T-Lymphocyte-Associated Protein 4 (CTLA-4), T-cell immunoglobulin and mucin domain-containing protein 3 (TIM-3), and natural killer cell group 2 member A (NKG2A) (110). These checkpoint molecules suppress CD8^+^ T cell proliferation, cytokine production (IFN-γ, granzyme B), and cytotoxic activity against ectopic endometrial cells.

The PD-1/Programmed cell death ligand 1 (PD-L1) axis is particularly well-characterized in EMs. In human studies, PD-1 expression is elevated on circulating CD8^+^ T cells and NK cells, while its ligands PD-L1 and PD-L2 are overexpressed on ectopic endometrial stromal cells, creating an immunosuppressive circuit that dampens cytotoxic clearance (111, 112). This immune checkpoint pathway directly impairs CD8^+^ T-cell mediated elimination of ectopic lesions.

In addition, gut microbiota-derived metabolites have been reported to inhibit V-domain Ig suppressor of T cell activation (VISTA) expression while enhancing CD8^+^ T-cell infiltration into endometriotic lesions, suggesting that the gut microbiome may influence the local immune checkpoint landscape (113). However, the clinical relevance of checkpoint blockade in EMs remains unclear and warrants careful investigation, given the substantial risk of immune-related adverse events in this predominantly reproductive-age population (100).

#### 4.2.3 B lymphocyte hyperactivation in endometriosis

B lymphocytes (B cells) serve dual core immune functions: antibody production and professional antigen presentation (114). Under physiological conditions, B cells constitute fewer than 5% of total tissue immune cells (115). Multiple clinical investigations report elevated B-cell counts, hyperactivated B-cell signaling, and excessive autoantibody production in EMs patients (116). The precise molecular mechanisms linking B-cell dysfunction to EMs pathogenesis remain incompletely characterized. Cumulative evidence indicates that aberrant B-cell expansion and hyperactivation enable endometrial cells to evade immune recognition, thereby accelerating disease progression. Systemic and local T-helper cytokine expression and lymphocyte activation are altered in EMs (117). Specifically, Antsiferova et al. demonstrated that both mRNA expression and intracellular synthesis of the Th2-type cytokines IL-4 and IL-10 were sharply increased in the peripheral blood of endometriosis patients, accompanied by a significant elevation of pan-B cells, CD20^+^CD5^+^B-1 cells, and activated HLA-DR^+^CD20^+^B lymphocytes in ectopic lesions. This Th2-skewed immune response directly promotes B lymphocyte proliferation and differentiation into plasma cells, while simultaneously suppressing cell-mediated immunity and removing the negative regulatory constraints on B-cell activation (100, 118). The resulting B-cell hyperactivation, in turn, drives autoantibody production and sustains chronic inflammation, forming a self-reinforcing circuit in which Th2 predominance and B-cell activation mutually amplify immune dysregulation and contribute to lesion persistence (100).

### 4.3 Humoral immunity and soluble immune mediators in endometriosis

#### 4.3.1 Defensins in endometriosis

Defensins are functional antimicrobial peptides that serve as core effectors of mucosal innate immunity. Both α-defensins and β-defensins have been well documented to mediate EMs pathogenesis (119). Chen et al. verified that the gene and protein expression of human β-defensin 2 (hBD-2) was significantly upregulated in ectopic endometrial tissues of EMs patients, compared with eutopic endometrial tissues from both EMs patients and healthy individuals (120). Correlation analyses further demonstrated that hBD-2 expression in both eutopic and ectopic endometrial tissues was positively associated with the levels of pro-inflammatory cytokines TNF-α and IL-1β (120). These findings indicate that TNF-α and IL-1β robustly induce hBD-2 upregulation, suggesting that the distinct inflammatory microenvironment within ectopic lesions drives excessive defensin expression. From the perspective of gut-immunity-metabolism crosstalk, gut dysbiosis-mediated chronic low-grade systemic inflammation elevates circulating TNF-α and IL-1β levels, thereby promoting defensin synthesis in the reproductive tract mucosa (13, 120). Beyond direct antimicrobial activity, defensins modulate microbial colonization patterns in the gut and reproductive tract. Dysregulated defensin secretion further disrupts reproductive tract microecological homeostasis, forming a self-amplifying positive feedback cascade: gut dysbiosis, systemic inflammation, defensin overexpression, and aggravated microecological imbalance (13, 121). Additionally, defensins exert critical immunomodulatory functions and actively reshape the immune microenvironment of endometriotic lesions. A clinical study involving 67 EMs patients and 16 healthy controls reported markedly elevated peritoneal fluid human neutrophil peptides 1-3 (HNP 1-3) levels in patients, which were positively correlated with disease severity (122). HNP 1–3 abundance was highly correlated with the counts of peritoneal neutrophils and T lymphocytes, as well as IL-8 concentrations. Given the confirmed T-cell chemotactic activity of HNP 1-3, defensins are speculated to participate in EMs immunopathogenesis via recruiting T lymphocytes and triggering local persistent inflammatory responses (123).

#### 4.3.2 Collectins in endometriosis

Collectins are soluble pattern-recognition receptors that specifically identify carbohydrate structures on the surface of pathogens and apoptotic cells (124). Mannose-binding lectin (MBL), a prototypical collectin molecule, activates the lectin complement pathway and facilitates apoptotic cell clearance (125). Although MBL and MASP-2 are detectable in the uterine mucosa of EMs patients, these molecules exhibit impaired biological activity within ectopic lesions (125). Importantly, patients with deficient lectin pathway function harbor a higher prevalence of pathogenic bacteria in the endometrial microbiome, indicating that impaired lectin signaling induces endometrial dysbiosis and further activates endometrial cellular inflammatory responses (125). However, clinical and mechanistic evidence supporting this regulatory axis remains limited. Moreover, a bidirectional regulatory relationship exists between MBL and estrogen. Estrogen modulates lectin pathway activity by regulating MBL expression, thereby remodeling local endometrial microecology. In turn, microbial metabolites such as SCFAs and LPS feedback to regulate local immune activation and hormonal signaling (125). The disruption of this homeostatic balance is recognized as a pivotal initiating event linking gut dysbiosis to pelvic inflammatory cascades in EMs.

#### 4.3.3 Complement system in endometriosis

The complement system is the most well-characterized humoral immune signaling pathway involved in EMs. As early as 1989, Isaacson’s team first demonstrated that *in vitro*-cultured ectopic endometrial cells can independently synthesize and secrete complement C3, providing direct evidence for complement involvement in EMs pathogenesis (126). Subsequent multi-omics studies have identified aberrant expression of multiple complement components in ectopic endometrial tissues, including C3, C4A, C7, CFD, CFB, and CFH, indicating concurrent activation of both classical and alternative complement pathways (127). Kabut et al. reported that EMs patients with reduced iC3b levels exhibited significantly increased concentrations of C3c, C4, and SC5b-9 in serum and peritoneal fluid, with distinct stage-specific expression patterns. Early-stage (Stage I-II) EMs is characterized by elevated iC3b, whereas advanced-stage (Stage III-IV) disease presents predominant SC5b-9 accumulation (128).

Complement cascade activation in EMs generates potent anaphylatoxins C3a and C5a, which bind to immune cell surface receptors and trigger robust local inflammatory reactions (100). C5a strongly induces the secretion of pro-inflammatory cytokines including IL-1β, IL-6, TNF-α, and IL-8 by peritoneal macrophages (129, 130). Furthermore, the membrane attack complex (MAC, C5b-9) assembled on ectopic cell membranes does not induce cell lysis; instead, it initiates intracellular pro-inflammatory signaling (128, 131). Sublytic MAC induces calcium influx, activates *NF-κB* and MAPK signaling cascades, and promotes the robust secretion of pro-inflammatory cytokines, adhesion molecules, and pro-survival factors (131, 132). Bacterial LPS infiltrating the bloodstream following intestinal barrier damage acts as an upstream trigger for complement activation, driving systemic and pelvic complement cascade activation and subsequent cellular metabolic reprogramming (133). Multi-omics analyses have further identified crosstalk between the complement and coagulation systems, which converges with metabolic signaling pathways such as JAK/STAT to amplify pelvic inflammation and tissue injury (134). Conversely, persistent chronic inflammation induced by excessive complement activation disrupts intestinal mucosal integrity, exacerbates gut dysbiosis, and ultimately forms a reciprocal pathological vicious cycle (100, 135). Notably, complement C3 serves as a core regulatory molecule throughout disease progression. *In vivo* animal experiments confirmed that C3-knockdown mice developed smaller endometriotic cysts and fewer ectopic lesions (136). Emerging studies focusing on alternative complement pathway components have revealed significantly elevated serum adipsin and complement factor H levels in EMs patients (137). Nevertheless, direct clinical evidence validating these findings remains insufficient, representing a promising direction for future EMs research.

#### 4.4 Systemic inflammatory microenvironment constructed by immune imbalance in endometriosis

Coordinated dysfunction across innate and adaptive immune compartments establishes a persistent systemic inflammatory microenvironment that underpins EMs progression. Innate immune failure permits initial lesion survival, adaptive immune dysregulation enables immune escape and chronic persistence, and their interaction sustains a self-reinforcing inflammatory circuit. Their interaction may sustain inflammatory signaling and provide a pathological niche associated with angiogenesis, fibrosis, and long-term lesion maintenance. These findings support immune imbalance as an important component of the multisystem pathophysiological features of EMs.

## 5 Host metabolic dysregulation in endometriosis: hallmark of multisystem dysfunction

### 5.1 Glucose metabolic reprogramming in endometriosis

Glucose metabolic reprogramming describes the adaptive rewiring of glucose utilization to meet the elevated energy and biosynthetic precursor demands of rapidly proliferating, invasive cells (138). Metabolic reprogramming is defined by a phenotypic shift away from mitochondrial oxidative phosphorylation as the primary ATP-generating pathway. Ectopic endometrial stromal cells preferentially utilize aerobic glycolysis for energy production, a phenotype marked by enhanced cellular glucose uptake, altered expression of glycolytic rate-limiting enzymes, and elevated lactate synthesis and transmembrane transport (139, 140).

Immunohistochemical profiling confirms widespread glucose transporter type 1 (GLUT1) and GLUT4 expression across ectopic lesions (141). The glycolytic enzymes lactate dehydrogenase A (LDHA) and pyruvate dehydrogenase kinase 1 (PDK1) are robustly upregulated in ectopic tissue. Elevated PDK1 activity inhibits pyruvate dehydrogenase (PDH), diverting accumulated pyruvate toward LDHA-mediated lactate production (142–145). LDHA expression is additionally modulated by local oxygen tension (146). Hypoxic peritoneal microenvironments further induce PDK1 expression to amplify glycolytic reprogramming (147).

Consequently, lactate concentrations are markedly elevated in both ectopic stromal cells and patient peritoneal fluid (142, 148). Lactate accumulation generates an acidic extracellular niche, mediates histone lactylation epigenetic modification, and may remodel immune cell functional profiles in ways that favor disease progression (148).

### 5.2 Lipid metabolic disorders in endometriosis

#### 5.2.1 Sphingolipid metabolism in endometriosis

EMs onset and progression are tightly coupled to dysregulated sphingolipid metabolism. Patient samples exhibit elevated phosphatidylcholine (PC) and sphingomyelin (SM) alongside reduced phosphatidylethanolamine (PE) concentrations (149). This lipid profile redirects metabolic flux from pro-apoptotic ceramide (Cer) toward mitogenic glucosylceramide (GlcCer), suppressing stromal cell apoptotic signaling (150). The Cer metabolite sphingosine-1-phosphate (S1P) accumulates within the peritoneal niche and has been associated with inflammation, angiogenesis, and macrophage M2 polarization (151).

Multiple independent studies validated SM and PC as candidate diagnostic biomarkers for EMs (152). Vouk and colleagues conducted targeted plasma metabolomic analyses encompassing 40 patients diagnosed with ovarian EMs and 52 healthy control individuals. The established diagnostic prediction model was based on the ratio of hydroxysphingomyelin to phosphatidylcholine. Following adjustment for age and BMI confounding factors, using stepwise logistic regression, the model yielded a diagnostic sensitivity of 90.0% and a specificity of 84.3%. Notably, this predictive model underwent only intra-cohort internal validation without external independent verification, and its applicability was strictly confined to ovarian EMs. Therefore, this lipid signature is currently recognized as a putative biomarker and necessitates rigorous prospective validation in large-scale, multicenter independent cohorts (152). This model provides a promising candidate biomarker panel approach for semi-invasive disease diagnosis.

#### 5.2.2 Cholesterol metabolism in endometriosis

*Homeobox A10* (*HOXA10*), a transcriptional suppressor of cholesterol biosynthesis, is markedly downregulated in ectopic endometrial stromal cells, resulting in enhanced *de novo* cholesterol synthesis (153). Intracellular cholesterol buildup is associated with increased activity of steroidogenic enzymes (cytochrome P450, family 11, subfamily A, polypeptide 1 (*CYP11A1*), *Cytochrome P450 family 17 subfamily A member 1* (*CYP17A1*), *Cytochrome P450 family 19 subfamily A member 1* (*CYP19A1*), driving local estradiol overproduction. Estradiol signaling upregulates prion protein expression, suppresses *peroxisome proliferator-activated receptor alpha* (*PPARα*) transcriptional activity, and induces 3-hydroxy-3-methylglutaryl-coenzyme A reductase (HMG-CoA), which may further increase cholesterol synthesis. Simultaneously, estradiol inhibits *ATP-binding cassette transporter A1* (*ABCA1*)-mediated cholesterol efflux from stromal cells, forming a potential self-amplifying estrogen-cholesterol positive feedback loop that sustains pathological lipid accumulation (154).

#### 5.2.3 Carnitine metabolism in endometriosis

Carnitine is an essential cofactor mediating mitochondrial fatty acid β-oxidation. Non-human primate EMs models demonstrate drastically reduced carnitine concentrations in ectopic lesions relative to eutopic endometrium, accompanied by depleted NADH, FAD, malate, and impaired mitochondrial respiratory control ratios (155). Vouk and colleagues performed targeted metabolomic profiling on peritoneal fluid samples collected from 29 patients with ovarian EMs and 36 healthy controls. The predictive model was constructed based on carnitine-to-phosphatidylcholine ratios and differential phosphatidylcholine abundance ratios. After age adjustment, using stepwise logistic regression, the model achieved a diagnostic sensitivity of 82.8%, a specificity of 94.4%, and an AUC value of 0.944. Consistent with its previously reported plasma counterpart, this model was internally validated within a small discovery cohort without independent external verification, and its applicability was strictly restricted to ovarian-type EMs. Collectively, this metabolic signature is currently defined as a putative biomarker. Rigorous multi-cohort validation is mandatory prior to any consideration of clinical translational application (156).

Conflicting reports exist regarding serum carnitine profiles across patient cohorts. Letsiou et al. identified a panel of circulating acylcarnitines predictive of EMs diagnosis (157). In contrast, Zhuang et al. reported elevated serum L-carnitine levels and proposed that *Streptococcus agalactiae* may promote *VEGF* expression through L-carnitine-associated metabolic alterations, thereby contributing to pathological angiogenesis (158). Further stratified cohort analysis is required to resolve these divergent observations.

In addition to fatty acid and carnitine metabolism, ketone body homeostasis is also disrupted in EMs. Notably, the ketone body 3-hydroxybutyrate, a product of fatty acid β-oxidation, is significantly elevated in the serum and follicular fluid of patients across both mild and severe stages. These consistent findings from independent clinical cohorts establish 3-hydroxybutyrate as a promising diagnostic biomarker (159).

### 5.3 Amino acid metabolic perturbations in endometriosis

Tryptophan metabolism exhibits a paradoxical dual regulatory pattern with coordinated metabolite fluctuations. Systemically, IL-1β elevates *tryptophan 2,3-dioxygenase* (*TDO2*) activity to accelerate tryptophan degradation; reduced tryptophan selectively induces Th1 apoptosis and shifts immunity toward Th2 dominance (160). Locally within ectopic lesions, *c-Jun N-terminal kinase* (*JNK*) signaling upregulates *indoleamine 2,3-dioxygenase 1* (*IDO1*), while estrogen further boosts *IDO1* transcription to facilitate Tregs’ differentiation (111, 112). Combined *TDO2* and *IDO1* overactivation leads to two prominent metabolite changes: global tryptophan depletion and robust kynurenine buildup, which jointly suppress the cytotoxic function of NK cells and effector T cells. Upregulated *IDO1* also modulates *Tumor protein p53* (*P53*), *Matrix metallopeptidase 9* (*MMP9*) and *Cyclooxygenase 2* (*COX2*) to elevate stromal proliferation and adhesion capacity (161). The tryptophan-kynurenine pathway is therefore proposed to contribute to immune evasion and lesion progression through distinct but overlapping downstream molecular cascades (111, 112).

EMs lesions display enhanced aerobic glycolysis that diverts pyruvate into lactate rather than mitochondrial TCA flux. To offset disrupted energy metabolism, ectopic stromal cells upregulate glutaminolysis, triggering prominent glutamine depletion in lesion tissues (161). Extracellular glutamine is transported into stromal cells via solute carrier family 1 member 5, catalyzed by glutaminase into glutamate, which is further converted to α-ketoglutarate. Elevated α-ketoglutarate replenishes TCA intermediates to sustain ATP production, biosynthetic precursor synthesis, mTOR activation and cellular redox homeostasis (162). Distinct from glutamine’s lesion-specific consumption, isoleucine is consistently downregulated across all analyzed EMs patient cohorts, serving as a universal marker of proliferation-adaptive amino acid remodeling (159).

Alanine shows consistent concentration decline in multiple patient biofluids. Serum alanine levels are markedly reduced, achieving 90% diagnostic sensitivity for stage I EMs (163, 164). When combined with other metabolite biomarkers, this alanine-centered signature reaches 100% sensitivity and 83% specificity for stage II EMs (163, 164). Patient follicular fluid also presents significantly lowered alanine concentrations, verifying the compartment-consistent hypoalaninemia phenotype of EMs (165).

Histidine and its derivative histamine display niche-specific divergent changes. Follicular fluid histidine concentrations are significantly elevated in EMs patients (166). High systemic histamine induces HDC transcriptional upregulation inside ectopic lesions; however, peritoneal fluid histamine concentrations are simultaneously suppressed, forming a unique bidirectional metabolic signature absent in other amino acid subgroups (167, 168). Despite independent upstream regulatory triggers and heterogeneous concentration shifts of individual amino acids, all four metabolites operate to facilitate two hallmark pathological phenotypes of EMs: (1) generating sufficient energy and biosynthetic substrates to sustain unlimited proliferation of ectopic stromal cells; (2) reshaping systemic and peritoneal immune microenvironment to achieve lesion immune evasion.

Glucose metabolism shifts toward aerobic glycolysis to fuel ectopic cell growth, lipid metabolism undergoes subtype-specific disruptions including altered sphingolipid pools, self-sustaining cholesterol overproduction and impaired fatty acid oxidation, while amino acid pathways display compartment-dependent metabolite fluctuations that jointly support lesion proliferation and immune suppression (**Figure 1**). Many altered metabolites across glucose, lipid and amino acid axes carry measurable diagnostic values for EMs staging, yet inconsistent circulating biomarker results from patient cohorts require further stratified clinical validation to unify conflicting metabolic observations (159).

**Figure 1:**
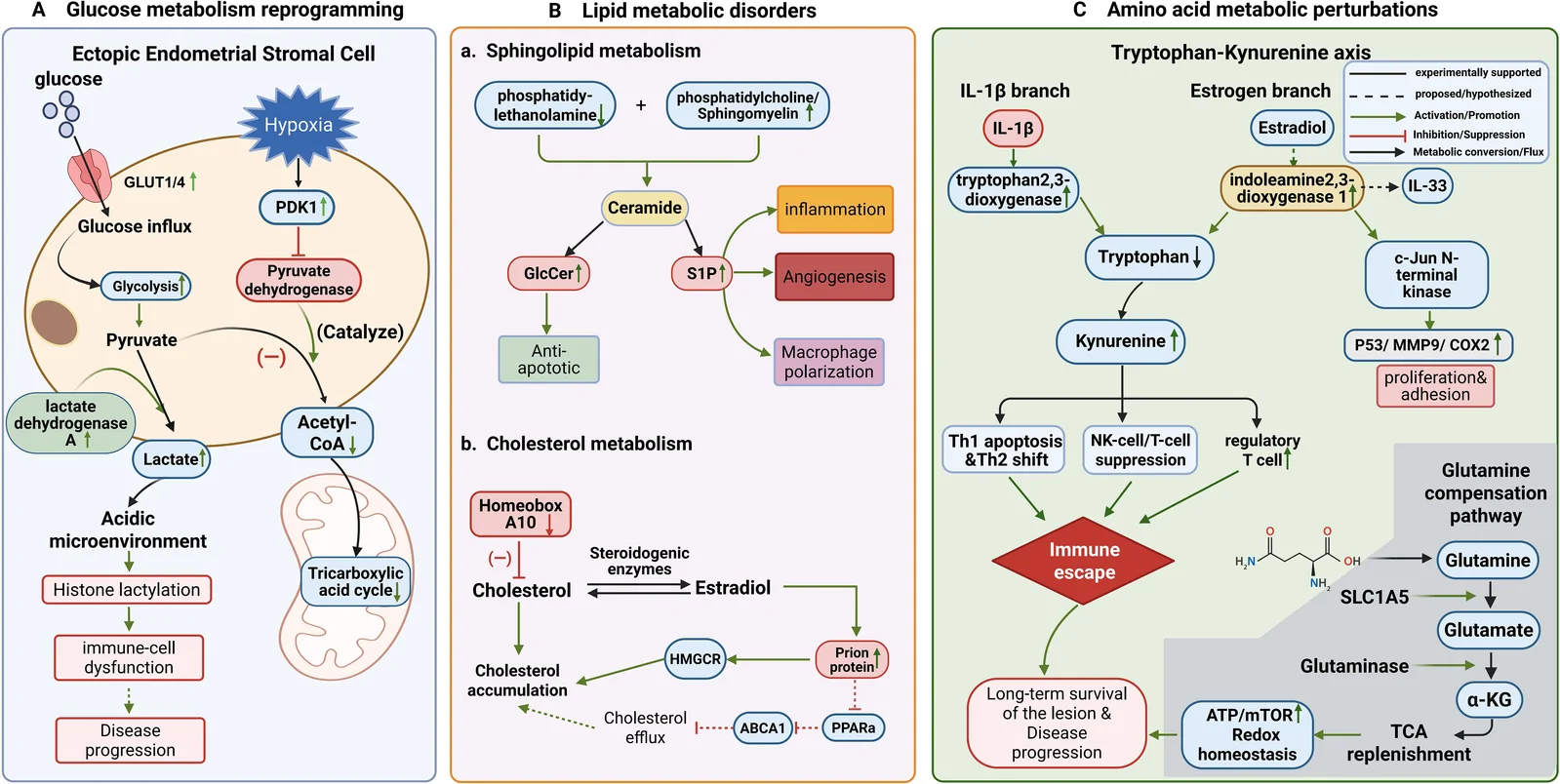
Host metabolic reprogramming in endometriosis. **(A)** The left panel depicts glucose metabolism reprogramming. Hypoxia drives pyruvate dehydrogenase kinase 1 (PDK1) upregulation, which inhibits pyruvate dehydrogenase (PDH) and blocks the conversion of pyruvate to acetyl-CoA, thereby limiting its entry into the tricarboxylic acid (TCA) cycle. Consequently, accumulated pyruvate is redirected toward lactate production via lactate dehydrogenase A (LDHA), leading to lactate accumulation, histone modification, and immune cell dysfunction, ultimately contributing to disease progression. **(B)** The middle panels summarize lipid metabolic disorders, including sphingolipid metabolism, cholesterol metabolism. **(a)** Elevated phosphatidylcholine (PC) and sphingomyelin (SM) and reduced phosphatidylethanolamine (PE) levels. Ceramide (Cer) is converted to two downstream metabolites: glucosylceramide (GlcCer), which promotes apoptosis resistance, and sphingosine-1-phosphate (S1P), which triggers peritoneal inflammation, angiogenesis and macrophage polarization. **(b)** Cholesterol metabolism showed homeobox A10 (HOXA10) downregulation leads to cholesterol accumulation and estradiol synthesis via steroidogenic enzymes (CYP11A1/CYP17A1/CYP19A1), forming a cholesterol-estradiol positive feedback loop; Peroxisome proliferator-activated receptor alpha: PPARα; 3-hydroxy-3-methylglutaryl-CoA reductase: HMGCR; ATP-binding cassette transporter A1:ABCA1. **(C)** The right panels show amino acid metabolic perturbations. The tryptophan-kynurenine axis via indoleamine 2,3-dioxygenase 1 (IDO1) and tryptophan 2,3-dioxygenase (TDO) drives tryptophan depletion and kynurenine accumulation. This leads to three parallel immune outcomes: Th1 apoptosis and Th2 shift, NK cell and T cell suppression, and regulatory T cell (Treg) expansion, all converging to immune escape. In parallel, IDO1 also activates the c-Jun N-terminal kinase (JNK) signaling pathway, leading to upregulation of P53, MMP9, and COX2, which promotes ectopic cell proliferation and adhesion. The glutamine compensation pathway via solute carrier family 1 member 5 (SLC1A5) supports tricarboxylic acid (TCA) cycle replenishment, adenosine triphosphate production, mammalian target of rapamycin activation, and redox homeostasis. All metabolic pathways converge to lesion survival, immune escape, invasion, angiogenesis, and disease progression. Arrow colors indicate the type of action: green arrows denote activation or promotion; red arrows denote inhibition or suppression; black arrows denote metabolic flux or conversion. Arrow styles indicate the level of evidence: solid lines denote pathways supported by experimental evidence (including human, animal, and cell-based studies); dashed lines denote proposed mechanisms or hypotheses that require further validation. All abbreviations are defined in this legend.).

## 6 Immunometabolic crosstalk: linking gut microbiota to multisystem pathophysiological features

### 6.1 Gut microbial metabolites in endometriosis: potential immune modulators bridging microbiota and immunity

The gut microbiome is a complex biological ecosystem. Bioactive compounds synthesized by enteric bacteria can enter systemic circulation, where they exert antiproliferative and anti-inflammatory effects (169). Among these, SCFAs, including butyrate, acetate, propionate, valerate, and caproate, primarily function by activating G protein-coupled receptors 43 (GPR43) and G protein-coupled receptor 109A (GPR109A) or by inhibiting histone deacetylases (HDACs) (170). EMs is increasingly recognized as having epigenetic characteristics; accordingly, HDAC1 expression is markedly upregulated in ectopic lesions, potentially promoting both lesion progression and fibrotic transformation (171). Microbial perturbation leads to a profound decrease in SCFAs, particularly affecting butyrate (172). Butyrate suppresses endometriotic lesion expansion through three distinct pathways: engagement of GPR43/GPR109A, inhibition of HDACs enzymatic activity, and activation of RAP1 GTPase-activating protein (RAP1GAP), which in turn inactivates RAP1 signaling (172). Furthermore, this metabolite modulates neutrophil function by inhibiting HDACs, thereby deactivating *NF-κB* and attenuating IL-1β signaling via NOD-like receptor family pyrin domain-containing 3 (NLRP3) inflammasome inhibition. This cascade effectively prevents cellular adhesion and proliferation (173, 174). The specific contributions of acetate, propionate, valerate, and caproate remain poorly characterized, representing a critical direction for future research.

The bacterial-derived indole metabolite 4-hydroxyindole (4HI) is significantly reduced in EMs patients, suggesting a potential protective role (175). In murine models, 4HI administration curbed infiltration of both peritoneal M1 and M2 macrophages, suppressed inflammation initiation and progression, and markedly alleviated pain responses (175). Notably, this treatment prevented lesion formation in a prophylactic model and induced substantial regression of established lesions in a therapeutic model (175). Collectively, these findings suggest that 4HI may exert immunomodulatory effects by mitigating inflammation, regulating immune cell infiltration, and providing analgesic benefits. However, these effects have not been established in human EMs.

In addition, Wang et al. reported significantly elevated 5-hydroxytryptamine (5-HT) levels in the peritoneal fluid of EMs patients, with Mendelian randomization analysis supporting a potential causal association between 5-HT and EMs risk, implicating the tryptophan, 5-HT metabolic axis as a potential contributor to disease progression (176). Meanwhile, Chen et al. identified *Parabacteroides goldsteinii* as a potential probiotic that is reduced in abundance in EMs patients and negatively correlates with disease severity; 7-ketolithocholic acid (7-KLCA) supplementation significantly reduced lesion burden, adhesion scores, and pain in mice (177). In a separate study, Yang et al. demonstrated that fluvastatin not only markedly reduced ectopic lesion volume and mass in mice but also reprogrammed lesion-infiltrating macrophages from the pro-lesional M2 phenotype toward the clearance-competent M1 phenotype, as evidenced by upregulated M1 markers (iNOS, CD86) and downregulated M2 markers (CD206, Arg1) (178).

### 6.2 Oxidative stress and redox signaling in endometriosis: reactive oxygen species-nuclear factor erythroid 2-related factor 2 pathway mediates microbiota-immune-metabolism crosstalk

Oxidative stress and dysregulated nuclear factor erythroid 2-related factor 2 (Nrf2) signaling are closely implicated in the pathological progression of EMs, and gut microbial dysbiosis may contribute to these alterations (179). Experimental studies have demonstrated that in an intestinal microbiota dysbiosis model, proliferation of opportunistic pathogens and subsequent release of LPS from their cell walls activate the TLR4 pathway, triggering excessive reactive oxygen species (ROS) production (180). Simultaneously, hemoglobin and free iron released from cyclic hemorrhagic events within lesions further catalyze ROS generation via the Fenton reaction (181).

Under physiological conditions, Nrf2 serves as the primary transcription factor orchestrating defense against oxidative damage, regulating expression of downstream antioxidant enzymes such as SOD, GPx, and HO-1 (182). In EMs, however, Nrf2 and its effector molecule GCL are significantly downregulated within lesions (182). Studies in Nrf2-knockout mice have shown that ectopic implants develop more pronounced fibrosis with increased volume and weight, confirming that Nrf2 inactivation directly potentiates lesion growth and fibrogenesis (182). The ensuing breakdown of antioxidant defenses causes persistent ROS accumulation, which simultaneously activates *NF-κB* and further represses Nrf2. This combined action increases ectopic cells’ ability to proliferate and invade (183).

Gut microbial metabolites play a critical role in redox regulation. 4HI exerts anti-inflammatory, antiproliferative, and analgesic effects in the experimental model. SCFAs modulate inflammatory signaling via HDACs inhibition and G-protein-coupled receptor activation (184). Reduced SCFAs availability may impair antioxidant and anti-inflammatory responses (184). Collectively, these findings support a potential link between gut microbial dysbiosis and ROS-Nrf2-mediated oxidative and inflammatory responses in EMs, although the contribution of gut-derived signals to this pathway requires further investigation.

### 6.3 Tricarboxylic acid cycle metabolic intermediates in endometriosis: regulating immune cell function and inflammatory microenvironment formation

Microbial imbalance may reshape TCA flux within peritoneal and circulating immune cells, altering the pool of intermediate metabolites and thereby modulating the chronic inflammatory milieu and lesion development. The TCA intermediate succinate has dual metabolic and signaling functions (185). Its signaling through the membrane-bound receptor *succinate receptor 1* (*SUCNR1*) is a principal mechanism driving establishment of a pro-inflammatory peritoneal environment (185).

Within the endometriotic peritoneal niche, M1-polarized macrophages accumulate and release substantial quantities of succinate due to a “break” in the TCA at the succinate dehydrogenase step; peritoneal mesothelial cells also contribute significantly to the local succinate pool (185). Concurrently, gut microbiome alterations disrupt the equilibrium between succinate-producing and succinate-consuming bacterial populations, leading to elevated intestinal succinate. This excess succinate can translocate to uterine tissues via the “gut–uterus axis”, further potentiating pelvic succinate accumulation (186).

Moreover, disrupted microbiota can directly secrete TCA metabolites such as succinate, activating immune cells through pattern recognition receptors. This interruption of the complete TCA promotes accumulation of pro-inflammatory metabolites, such as succinate and fumarate, while also engaging the itaconate shunt. Extracellular succinate thus acts as an “inflammatory alarm” signal: it activates myeloid cells via *SUCNR1* and stabilizes HIF-1α by inhibiting α-ketoglutarate-dependent dioxygenases. This signaling cascade subsequently activates *NF-κB* and the NLRP3 inflammasome, promoting release of pro-inflammatory cytokines and exacerbating local inflammation and immune dysregulation. In contrast, itaconate and α-ketoglutarate exert compensatory anti-inflammatory effects, and may antagonize succinate-associated inflammatory signals. The balance between these metabolites may modulate macrophage polarization and T-cell differentiation, leading to alterations in lesion proliferation, immunoregulation, and disease progression. Dysregulated gut microbiota reshapes systemic bioactive metabolites including SCFAs, 4HI and succinate, which translocate through the gut–uterus axis to control peritoneal immunity, macrophage polarization and ectopic lesion expansion via epigenetic, inflammatory and redox signaling cascades. Microbial imbalance also fuels persistent oxidative stress through the ROS-Nrf2 vicious cycle and disturbs immune cell TCA homeostasis, jointly amplifying pelvic inflammation, fibrogenesis and endometriotic pathological progression (**Figure 2**).

**Figure 2:**
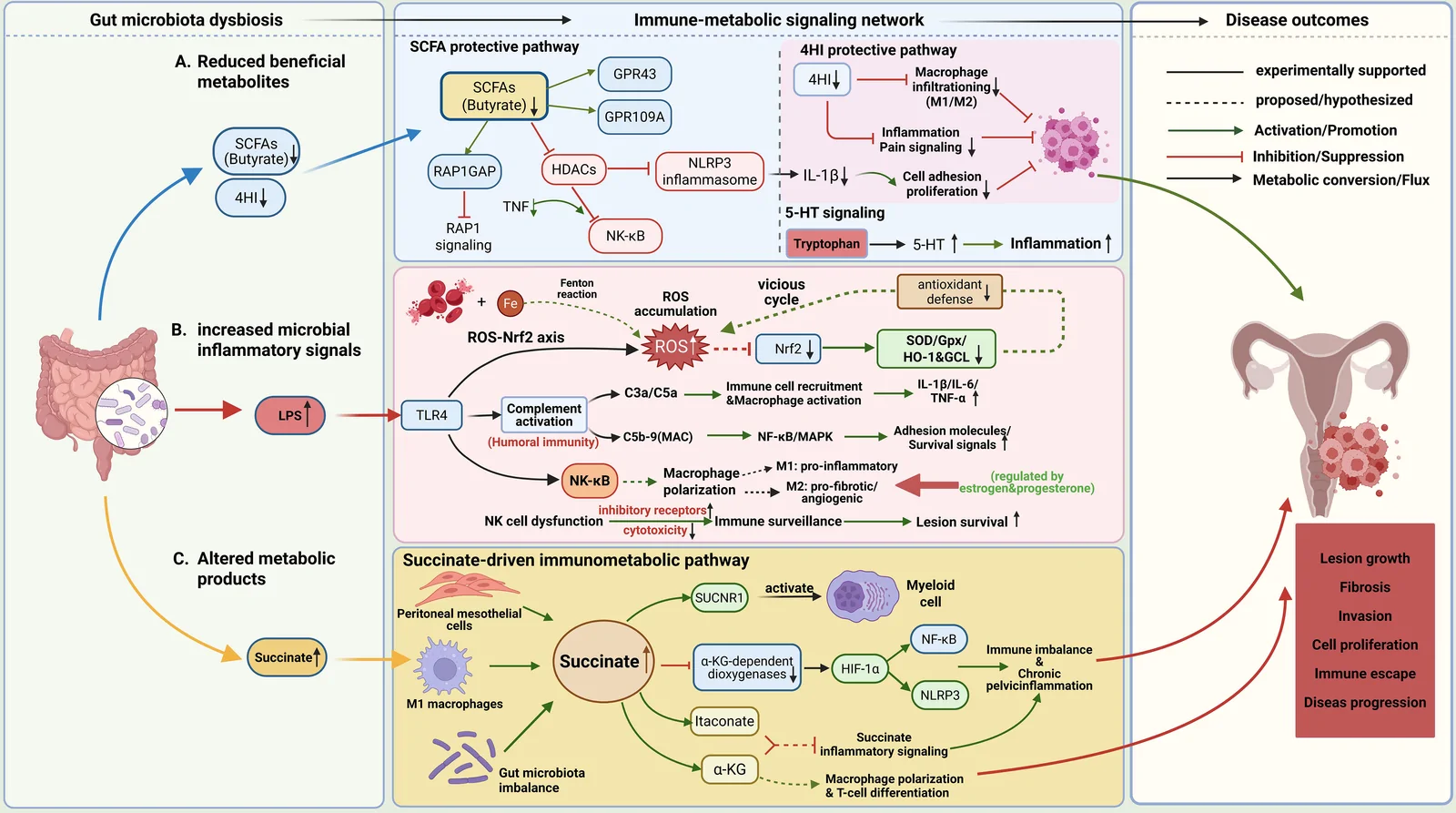
Immunometabolic crosstalk linking gut microbiota, immune dysregulation, and metabolic perturbation in endometriosis. **(A)** Gut microbial metabolites mediate gut-uterus immune regulation in endometriosis. Dysbiosis lowers protective short-chain fatty acids (SCFAs) especially butyrate. Butyrate restrains lesions via G protein-coupled receptor 43 (GPR43)/G protein-coupled receptor 109A (GPR109A), histone deacetylase (HDAC) suppression and RAP1 GTPase-activating protein (RAP1GAP)-mediated RAP1 inhibition, blocking nuclear factor kappa-light-chain-enhancer of activated B cells (NF-κB)/NOD-like receptor family pyrin domain-containing 3 (NLRP3)/interleukin-1β (IL-1β) signals. Patient 4-hydroxyindole (4HI) declines; this indole metabolite limits peritoneal macrophage infiltration, relieves inflammation and pain, and reduces ectopic lesions in mouse models. Functions of other SCFAs subtypes require further exploration. **(B)** The reactive oxygen species (ROS)-Nrf2 axis links gut dysbiosis to endometriosis progression. Pathogen-derived LPS and lesion iron jointly boost ROS via TLR4 and Fenton reactions. Lesion Nrf2 and its antioxidant targets are suppressed, amplifying NF-κB and ectopic cell invasion. Deficient SCFAs weaken Nrf2-mediated protection, while microbial 4HI relieves oxidative injury. Targeting this redox axis holds therapeutic potential. In addition, the LPS-TLR4 axis engages two interconnected effector branches: the complement system (C3/C5a and C5b-9) as part of humoral immunity, and NF-κB-driven macrophage polarization (M1/M2 imbalance) contributing to inflammation, fibrosis, and angiogenesis. Natural killer (NK) cell dysfunction, characterized by upregulated inhibitory receptors and reduced cytotoxicity, further impairs immune surveillance and promotes lesion survival. Macrophages are also modulated by hormonal signals, including estrogen and progesterone. **(C)** Gut dysbiosis disturbs immune cell tricarboxylic acid (TCA) cycling and drives succinate accumulation via the gut–uterus axis. Macrophages and mesothelial cells secrete succinate. Succinate binds SUCNR1, stabilizes hypoxia-inducible factor 1-alpha HIF-1α, and activates NF-κB and NLRP3 inflammasomes to boost pro-inflammatory cytokine release. Pro-inflammatory succinate and fumarate counteract anti-inflammatory itaconate and α-ketoglutarate (α-KG); their balance shapes immune cell differentiation and lesion growth. Combined with SCFAs/4HI loss and ROS-Nrf2 redox dysfunction, this metabolic disorder accelerates endometriosis progression. Arrow colors indicate the type of action: green arrows denote activation or promotion; red arrows denote inhibition or suppression; black arrows denote metabolic flux or conversion. Arrow styles indicate the level of evidence: solid lines denote pathways supported by experimental evidence (including human, animal, and cell-based studies); dashed lines denote proposed mechanisms or hypotheses that require further validation. All abbreviations are defined in this legend.

## 7 Clinical implications: biomarkers and therapeutic strategies targeting the microbiome-immune-metabolic axis

### 7.1 Multi-omics combined biomarkers in endometriosis: non-invasive diagnostic system based on microbiome and metabolome

In metabolomics studies, fecal analysis has identified 22 EMs-specific metabolites, 12 of which show potential as non-invasive fecal diagnostic markers (187). Serum and plasma metabolomics have revealed 26 potential diagnostic metabolites; a classification model integrating autoantibodies achieved a sensitivity of 0.98 and a specificity of 0.86 (30). A recent preprint study reported a distinct serum androgen profile in 159 patients with EMs compared with 57 healthy controls, suggesting the potential utility of steroid metabolomic profiling for EMs diagnosis (188). Urinary nuclear magnetic resonance -based metabolomics can identify candidate biomarkers including threonic acid, 3-hydroxybutyrate, succinate, citrate, lactate, and acetone (159).

Microbiome profiling also offers promise for early non-invasive diagnosis of EMs (20). In a prospective cohort study, Hicks et al. identified significant enrichment of oral Fusobacterium in patients with moderate-to-severe EMs (189). *Fusobacterium nucleatum* (*F. nucleatum*), a commensal microbe colonizing the human oral cavity and intestinal tract, has been detected in endometrial tissues from up to 64% of women diagnosed with EMs. Functional experimental evidence confirms that *F. nucleatum* infection activates the TGF-β signaling cascade in endometrial cells, thereby driving the transdifferentiation of fibroblasts into transgelin-positive myofibroblasts. These transformed myofibroblasts possess enhanced proliferative, adhesive, and migratory capabilities. Multiple bacterial components, including microbial metabolites (formate, lactate, hydrogen sulfide), virulence proteins (FadA, Fap2), and lipid mediators (oxidized low-density lipoprotein, lysophosphatidylcholine), cooperatively trigger local inflammatory infiltration, immune evasion, and epithelial-mesenchymal transition. Host pathological conditions, including tissue hypoxia, systemic estrogen predominance, and retrograde menstruation, collectively create a favorable pathogenic niche that supports *F. nucleatum* colonization and potentiates its virulence. Collectively, these findings demonstrate that oral-derived *F. nucleatum* serves as both a promising non-invasive diagnostic biomarker and a critical upstream pathogenic agent capable of translocating from the oral cavity to the female reproductive tract. An integrated microbiome-metabolome analysis of pelvic lavage fluid further validated that *Pseudomonas* enrichment constitutes a potential pathogenic driver of EMs. Increased *Pseudomonas* abundance is strongly correlated with enlarged ectopic lesions, severe pelvic adhesion, and advanced-stage (Stage III-IV) disease (190). Intestine-derived *Pseudomonas* (*P. aeruginosa*) induces LPS-mediated neutrophil extracellular trap (NET) formation in a unique MME^+^ neutrophil subset in response to bacterial LPS stimulation. The accumulated LPS directly binds and traps endometrial cells, facilitates their proliferation and migration, and ultimately propels continuous ectopic lesion progression (191).

Overall, these findings indicate that metabolomic and microbiome profiling may provide complementary approaches for EMs diagnosis. As summarized in **Table 2**, candidate biomarkers have been identified across multiple biological compartments, including feces, serum, plasma, urine, peritoneal or pelvic lavage fluid, oral samples, and endometrial tissue (192). Several metabolomic signatures have demonstrated relatively high diagnostic sensitivity and specificity, whereas microbiome-based markers remain largely exploratory. Importantly, most currently available biomarker models have been developed in single-center or discovery cohorts and lack independent external validation, highlighting the need for larger prospective and multicenter studies before clinical implementation (193).

**Table 2:** Diagnostic applications of metabolomics and microbiome profiling.

Biomarker	Sample type	Platform	Cohort size	Disease stage	Sensitivity	Specificity	External validation	Ref
SM/PC ratio panel	Plasma	Targeted metabolomics	40 EMs/52 controls	Ovarian EMs	90.0%	84.3%	No (discovery cohort only)	(152)
Carnitine/PC ratio (C0/PC ae C36:0)	Peritoneal fluid	Targeted metabolomics	29 EMs/36 controls	Ovarian EMs	82.8%	94.4%	No (discovery cohort only)	(156)
26 serum/plasma metabolites + autoantibodies	Serum/plasma	Metabolomics	NR	NR	98.0%	86.0%	No (classification model)	(129)
3-hydroxybutyrate	Serum/follicular fluid	NMR-based metabolomics	Multiple cohorts (systematic review)	Mild and severe	NR	NR	Yes (multiple independent cohorts)	(100)
Urinary metabolites (threonic acid, succinate, citrate, lactate, acetone)	Urine	NMR-based metabolomics	NR	NR	NR	NR	No (candidate biomarkers)	(100)
Fusobacterium (oral)	Oral samples	Microbiome sequencing	Prospective cohort	Moderate-severe EMs	NR	NR	No (single cohort)	(189)
Pseudomonas (pelvic lavage)	Pelvic lavage fluid	Microbiome sequencing	NR	Stage III-IV	NR	NR	No (single cohort)	(190)

*NR, not reported; EMs, endometriosis; SM, sphingomyelin; PC, phosphatidylcholine; C0, free carnitine; ae, acyl-alkyl (lipid subclass); NMR, nuclear magnetic resonance.

Despite encouraging preliminary results, substantial heterogeneity remains across studies with respect to sample source, analytical platform, disease subtype, disease stage, and cohort composition. Future studies should therefore prioritize standardized analytical workflows, independent external validation, and integrated multi-omics approaches to determine whether combined metabolomic and microbiome signatures can provide robust and clinically applicable diagnostic tools for EMs.

### 7.2 Microbiome-targeted intervention strategies

Diet is well-established as a factor influencing EMs (194). In terms of nutritional therapies, diets high in polyunsaturated fatty acids (PUFAs) (found in fish and nuts) along with an abundance of fruits, vegetables, and dairy products are linked to reduced symptoms and a decreased risk of EMs (195, 196). Conversely, high consumption of trans fats, red meat, and alcohol is associated with increased risk (31). Nevertheless, it is clinically pivotal to rigorously differentiate symptomatic relief and quality-of-life improvements from direct modulatory effects on endometriotic lesions, as these two categories represent fundamentally distinct therapeutic endpoints.

The Mediterranean Diet (MedDiet) focuses on plant-based foods with olive oil as the main fat source. It restricts animal protein intake to moderate amounts of poultry, fish, and dairy, with total fat contributing no more than 30% of energy and saturated fat at 8-10% or less. This diet’s bioactive ingredients lower inflammation indicators including IL-6 and TNF-α, leading to pain relief over time (197). Available evidence supports only symptomatic relief and mitigation of systemic inflammation, whereas direct evidence for endometriotic lesion regression remains lacking.

The Ketogenic Diet (KD), a regimen with minimal carbohydrate, moderate protein, and high fat intake, inhibits the NLRP3 inflammasome via β-hydroxybutyrate (BHB), downregulating key pro-inflammatory cytokines including IL-1β, TNF-α, and IL-6. This creates an anti-inflammatory environment and enhances mitochondrial function (198, 199). Available evidence supports only symptomatic improvement and reductions in systemic inflammatory markers, but it remains to be determined whether the anti-inflammatory properties of this dietary intervention can genuinely promote endometriotic lesion regression.

The Low FODMAP Diet restricts fermentable oligosaccharides, disaccharides, monosaccharides, and polyols to minimize intestinal gas production and osmotic load (198, 200). The EndoFOD randomized controlled cross-over trial demonstrated that 21 of 35 subjects (60%) responded to a 28-day low-FODMAP dietary regimen, whereas only 9 of 35 subjects (26%) in the control group achieved a therapeutic response (P = 0.008). Additionally, patients exhibited significant improvements in abdominal pain, bloating, stool consistency, and overall quality of life (201). These findings provide preliminary clinical evidence supporting the capacity of low-FODMAP diets to ameliorate EMs-related gastrointestinal symptoms, though further validation in large-scale, well-designed studies is still warranted. Of note, these benefits stem predominantly from relief of gastrointestinal IBS-like symptoms and not direct biological actions against ectopic lesions.

Among microbiota-targeted therapies, probiotics can modulate β-glucuronidase activity, influencing estrogen levels and sustaining immune homeostasis through anti-inflammatory responses (202, 203). Notably, *Lactobacillus gasseri OLL2809* inhibits development of ectopic endometrial cells in the peritoneal cavity by activating NK cells (204). In experimental murine models of EMs, a Western-style diet increased lesion volume, accompanied by a significant decline in the abundance of *Akkermansia muciniphila* (*A. muciniphila*) (205). These observations indicate a meaningful correlation between dietary patterns, *A. muciniphila* abundance, and endometriotic lesion progression. Recent preclinical data further reveal that oral supplementation with *A. muciniphila* can reduce the volume and weight of endometriotic lesions in mice (178). Notably, these promising findings are exclusively derived from animal models and require rigorous validation in human clinical studies.

In a related clinical study, a one-month probiotic intervention involving 20 EMs patients induced multiple molecular alterations in endometrial lesions, most notably, a significant upregulation of NLRP3 inflammasome mRNA expression. These findings suggest that probiotics may influence local immune microenvironment remodeling, but the clinical significance and causal mechanisms remain uncertain (206).

Fecal microbiota transplantation (FMT) remains an experimental approach. Studies indicate that FMT from healthy donors may remodel the gut microbiota, elevate acetate levels, and reinforce the intestinal barrier. In human studies, with reported improvements in disease-related outcomes. In contrast, patient-derived FMT disrupts the barrier and promotes bacterial translocation to the peritoneal cavity (191, 207).These findings provide preclinical support for microbiota-mediated effects but do not establish efficacy in patients with EMs.

### 7.3 Immune system-targeted intervention strategies

Rather than representing independent therapeutic options, current treatment strategies for EMs reflect the progressive evolution of disease concepts, from an estrogen-dependent disorder to a chronic inflammatory disease, and more recently to an immune-mediated condition characterized by immune dysfunction and immune escape. This paradigm shift has fundamentally reshaped therapeutic development, with increasing emphasis on restoring immune homeostasis rather than simply suppressing lesion growth or inflammation.

Hormonal therapy remains the clinical cornerstone because estrogen is a principal factor associated with lesion survival and inflammatory activation. Progestins are recommended as first-line agents owing to their combined anti-estrogenic and immunomodulatory properties (208). Beyond inhibiting ectopic lesion proliferation, progestins suppress macrophage activation, reduce the production of IL-1β, IL-6, monocyte chemoattractant protein-1 (MCP-1), and TNF-α, inhibit both classical and alternative macrophage polarization, and partially restore NK-cell cytotoxicity (209). Similarly, GnRH agonists and aromatase inhibitors reduce endogenous estradiol synthesis, thereby attenuating estrogen-dependent inflammatory signaling (210). However, increasing evidence suggests that hormonal suppression alone rarely eliminates established lesions because it may not fully reverse the self-sustaining inflammatory and immunosuppressive microenvironment that persists after disease initiation.

Recognition of chronic inflammation as a key pathogenic feature has supported the development of cytokine-targeted therapies. Persistent activation of TNF-α, IL-1β, IL-6, and IL-17 provides a rationale for targeting these inflammatory mediators (92). JAK inhibitors, which have demonstrated efficacy in autoimmune disorders, are emerging as promising candidates for suppressing multiple cytokine signaling pathways simultaneously (136). Likewise, TNF-α inhibitors and IL-1 receptor antagonists may alleviate symptoms by dampening inflammatory cascades (211). Nevertheless, clinical translation has been limited because these approaches primarily neutralize downstream inflammatory mediators without correcting the underlying immune-cell dysfunction that may contribute to sustained cytokine production.

This limitation has shifted attention toward immune cell-based therapies that may directly restore immune surveillance. Experimental studies have demonstrated proof-of-concept for macrophage and NK-cell depletion or adoptive transfer, with effects on disease progression in animal models (212, 213). More recently, therapeutic strategies have evolved from immune-cell depletion toward functional reprogramming. Macrophage repolarization, blockade of inhibitory NK-cell receptors to enhance cytotoxicity, and infusion of *in vitro*-activated autologous immune cells have shown encouraging efficacy in preclinical studies (212, 214). This transition reflects a broader conceptual shift in immunotherapy, recognizing that immune cells exhibit remarkable functional plasticity and that restoring immune competence may be more advantageous than indiscriminate immune depletion. However, these approaches remain experimental, and their long-term safety, durability, and reproductive consequences require rigorous validation in well-designed clinical trials.

Growing evidence that endometriotic lesions actively evade immune elimination has further expanded therapeutic possibilities. Aberrant expression of immune checkpoint molecules, including PD-1/PD-L1, CTLA-4, TIM-3 and NKG2A, has been identified in lesions, immune cells, serum, and peritoneal fluid (110). Moreover, ectopic stromal cells establish an immunosuppressive niche through the expression of PD-L1/2, FasL, CD47, and related immune-regulatory molecules (215), suggesting that EMs shares key mechanisms of immune escape with malignancies. Consequently, immune checkpoint blockade has emerged as a compelling experimental strategy. However, unlike oncological malignancies, EMs is a benign disorder that predominantly affects reproductive-age women with fertility aspirations, which fundamentally alters the risk-benefit calculus. Major safety concerns include: reproductive toxicity compromising ovarian reserve and oocyte quality (216); pregnancy-associated risks, including fetal rejection and developmental toxicity (217); immune-checkpoint-inhibitor-associated autoimmune adverse events, which would be unacceptable in otherwise healthy young women (218); systemic immune overactivation that may unmask pre-existing subclinical autoimmunity (218); and incompletely characterized long-term effects on immune and reproductive health (216). Accordingly, immune checkpoint blockade remains purely a hypothesis-generating experimental avenue, and translational research may only be contemplated once robust human safety data have been accumulated.

Conventional anti-inflammatory therapies continue to provide symptomatic benefit. NSAIDs alleviate pain by reducing prostaglandin synthesis through COX-1/2 inhibition and may suppress angiogenesis by attenuating *VEGF* signaling (219). Recent evidence further indicates that NSAIDs partially reverse macrophage-mediated T-cell suppression, highlighting previously underappreciated immunomodulatory effects (220). Furthermore, emerging epigenetic therapies, exemplified by Bobcat339, which promotes degradation of overexpressed *Tet methylcytosine dioxygenase 3* (*TET3*), represent a novel strategy for simultaneously regulating inflammatory and immune pathways (221).

### 7.4 Metabolic modulation therapy: a novel intervention direction for endometriosis

An emerging concept that is reshaping the field is that immune dysfunction may be influenced by metabolic reprogramming. Increasing evidence indicates that glycolysis, lipid metabolism, amino acid metabolism, and lactate accumulation are not merely metabolic adaptations supporting lesion growth but also upstream regulators of macrophage polarization, NK-cell dysfunction, T-cell exhaustion, and myeloid-derived suppressor cell expansion. From this perspective, metabolic dysregulation constitutes the mechanistic bridge linking endocrine abnormalities, chronic inflammation, and immune dysfunction. Accordingly, metabolic regulatory therapy has evolved from a lesion-centered intervention into a strategy for immunometabolic reprogramming. Several promising approaches have been reported. Ginsenoside Rg3 suppresses glycolysis by regulating the TRIM28/PDK4 axis; metformin exerts synergistic therapeutic effects through improving insulin sensitivity and metabolic homeostasis; nanodelivery systems targeting the lactate-MDSC axis simultaneously remodel metabolic and immune microenvironments; and resveratrol reduces lesion burden by modulating lipid metabolism and activating *PPARα* (22, 222–224). However, these findings are largely preclinical, and metabolic pathways are highly heterogeneous among patients and are closely integrated with systemic physiology, making target selection, biomarker identification, and long-term safety major challenges for clinical translation.

Multi-omics microbiome and metabolome signatures enable non-invasive EMs diagnosis, while layered intervention strategies covering dietary microbiota regulation, immune-targeted agents and immunometabolic modulators are developed to reverse lesion-supporting inflammatory, immune and metabolic abnormalities yet face translational limitations including insufficient clinical validation and patient heterogeneity barriers.

Importantly, the therapeutic strategies illustrated in **Figure 3** differ markedly in the strength of supporting evidence, distinguishing well-established clinical therapies from investigational interventions. Category A includes well-established clinical therapies, including hormonal treatments and NSAIDs, which represent first-line standard-of-care options for symptom control and lesion suppression. Category B covers interventions supported by preliminary or emerging clinical evidence, including dietary modification and probiotic supplementation. Category C consists of interventions predominantly backed by pre-clinical animal-model data, such as FMT, immune-cell-based therapies, metabolic modulators, and nanomedicine drug-delivery systems. Category D encompasses interventions confined to *in-vitro* experiments or theoretical conceptual frameworks, such as epigenetic drugs, and resides at the very earliest stages of therapeutic development.

**Figure 3:**
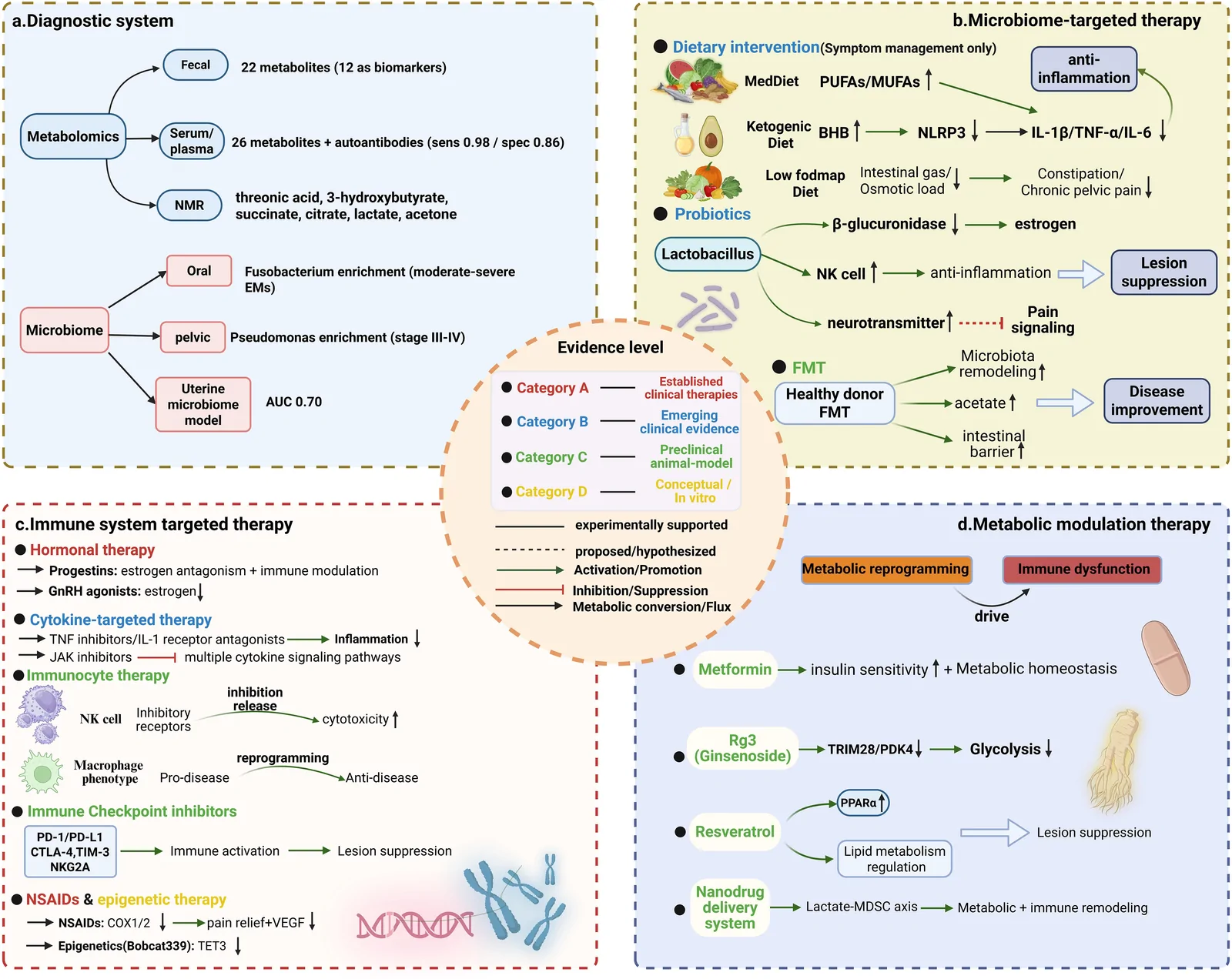
Systems-level therapeutic strategies targeting the gut-lesion immunometabolic axis. **(a)** Diagnostic system is based on multi-omics biomarkers, including metabolomics and microbiome profiling, for non-invasive diagnosis. Nuclear magnetic resonance: NMR; Area under curve: AUC; **(b)** Microbiome-targeted therapy includes dietary intervention of Mediterranean diet (Med Diet), ketogenic diet, and low FODMAP Diet, probiotics, and fecal microbiota transplantation (FMT). **(c)** Immune system-targeted therapy contains hormonal therapy, immunocyte therapy, immune checkpoint inhibitors targeting programmed cell death protein 1 (PD-1) and its ligand 1 (PD-L1), cytotoxic T-lymphocyte-associated protein 4 (CTLA-4), T-cell immunoglobulin and mucin domain-containing protein 3, and natural killer cell group 2 member A (NKG2A), non-steroidal anti-inflammatory drugs (NSAID) and epigenetic therapy. **(d)** Metabolic modulation therapy consists of metformin, ginsenoside Rg3, resveratrol, and nanodrug delivery systems targeting the lactate-myeloid-derived suppressor cell axis. All four therapeutic approaches converge to lesion suppression and disease improvement. Color-coded labels represent four levels of evidence according to the manuscript text: red (Category A), established clinical therapies; blue (Category B), evidence from clinical trials or emerging clinical studies; green (Category C), preclinical evidence based on animal models; yellow (Category D), *in vitro* or conceptual evidence. For dietary interventions, available evidence supports symptom relief rather than direct pathological lesion suppression. Arrow colors indicate the type of action: green arrows denote activation or promotion; red arrows indicate inhibition or suppression; black arrows denote metabolic flux or conversion. Arrow styles indicate the level of evidence: solid lines mark pathways supported by experimental data (human, animal, and cell-based studies); dashed lines mark proposed mechanisms or hypotheses awaiting further validation. All abbreviations are defined in this legend.).

## 8 Challenges and future directions

Accumulating multi-omics evidence indicates widespread gut microbial dysbiosis, disturbed glucose-lipid-amino acid metabolism and impaired immune homeostasis across EMs cohorts, but critical knowledge gaps hinder clinical translation. Although gut microbiota, microbial metabolites and redox-TCA signaling axes are proven to mediate gut-uterus crosstalk driving lesion proliferation and immune evasion, high-quality causal evidence verifying their direct pathogenic roles remains insufficient (225). EMs exhibits prominent interpatient and lesion-subtype heterogeneity, and existing preclinical animal models fail to fully recapitulate complex human metabolic, microbial and inflammatory phenotypes, weakening the generalizability of mechanistic findings. For diagnosis, numerous microbiome and metabolome candidate markers from serum, urine and feces have been uncovered, yet no single analyte satisfies clinical sensitivity and specificity standards; integrated multi-omics biomarker panels still lack cross-cohort validation (30). Current therapeutic strategies including hormonal drugs, anti-inflammatory agents, probiotic supplements and metabolic regulators only relieve partial symptoms, as they cannot reverse the self-amplifying cycle of dysbiosis, metabolic reprogramming and immune suppression, resulting in high recurrence rates after treatment.

To resolve these intertwined bottlenecks, standardized multi-dimensional research systems are urgently needed. Standardized protocols should be implemented for sampling, sequencing, metabolomic profiling, and clinical phenotyping, with rigorous control over confounding variables including menstrual cycle phase, diet, medication history, age, BMI, disease stage, lesion subtype, prior surgical history, and sample processing. Then, researchers ought to combine harmonized longitudinal multicenter multi-omics data with Mendelian randomization analysis to solidify causal links between gut microbial metabolites, immunometabolic disruption and EMs progression. Correlative clinical observations require functional verification via optimized organoid and primate models before cross-population replication. Multi-compartment non-invasive biomarker signatures should be refined to distinguish superficial, ovarian and deep infiltrating EMs for early screening and precise patient stratification. Finally, large-scale multicenter randomized controlled trials are required to evaluate microbiota-targeted diets, probiotics and FMT, alongside immunotherapies and metabolic modulators, to develop precision combinatory regimens targeting the microbiota-metabolism-immune axis.

## 9 Conclusion

This review synthesizes evidence demonstrating that gut microbial dysbiosis, immune dysfunction, and host metabolic disturbance serve as interconnected components of the multisystem pathophysiological characteristics of EMs. Alterations in gut microbiota composition, estrogen metabolism, intestinal barrier function, immune regulation, and metabolic reprogramming can interact across multiple biological levels; yet, the strength of available evidence varies across distinct pathways and study designs. Human observational studies largely support these associative findings, while genetic analyses, animal models, and cellular experiments provide complementary evidence for specific mechanistic pathways. Notably, most observed gut microbe–endometriosis associations reported to date remain correlative, without definitive evidence of causality from human studies (225). Furthermore, reverse causal interplay is non-negligible: EMs itself can reshape gut microbial composition through multiple pathological pathways, including pain-induced dietary pattern alterations, frequent clinical administration of NSAIDs, hormonal therapies and antibiotics, chronic stress-mediated hypothalamic-pituitary-adrenal (HPA) axis hyperactivation, and abnormal intestinal motility (225–227). Most existing microbiome investigations adopt cross-sectional observational designs, which cannot disentangle causal directionality (69). Accordingly, gut dysbiosis may serve as a pathogenic trigger, a secondary disease consequence, a key disease-modifying regulator, or merely an innocent epiphenomenon of EMs. Most plausibly, these scenarios coexist synergistically to form a dynamic reciprocal pathological loop. Large-scale longitudinal cohort studies and microbiota-targeted interventional trials are urgently required to clarify the definitive role of gut microbial disturbance in EMs pathogenesis and progression (60). Based on the current body of evidence, gut microecological disorders and EMs should be defined as a dynamic, bidirectional, cascade-driven pathological loop, rather than a simplistic unidirectional linear relationship whereby microbiota dysbiosis induces EMs. This updated cognitive framework establishes rigorous theoretical and methodological boundaries for future precision-oriented, time-resolved microbially targeted diagnosis and individualized treatment strategies (228).

Complementary immunometabolic evidence further identifies synchronized reshaping of innate and adaptive immunity together with widespread reprogramming of glucose, lipid and amino acid metabolism; this coordinated molecular cascade jointly maintains ectopic cell survival, enables immune evasion, stimulates pathological angiogenesis and accelerates pelvic fibrosis. The tripartite interaction model, comprising gut microbiota dysbiosis, immune dysregulation, and host metabolic reprogramming, constructed in this work delivers unified theoretical logic to reconcile previously scattered findings from microbiomic, immunological and metabolic investigations of endometriosis. The conceptual framework and its mechanistic interconnections are illustrated in **Figures 1** and **2**. All non-invasive multi-omics diagnostic candidates and multi-tiered intervention regimens, including dietary modulation, microbiota-targeted therapy, immune checkpoint treatment and metabolic reprogramming agents, gain clear pathogenic rationales under this gut–uterus axis paradigm.
